# A systematic literature review: deep learning techniques for synthetic medical image generation and their applications in radiotherapy

**DOI:** 10.3389/fradi.2024.1385742

**Published:** 2024-03-27

**Authors:** Moiz Khan Sherwani, Shyam Gopalakrishnan

**Affiliations:** Section for Evolutionary Hologenomics, Globe Institute, University of Copenhagen, Copenhagen, Denmark

**Keywords:** deep learning, convolutional neural network, radiotherapy, synthetic CT, photon therapy, proton therapy, generative adversarial network

## Abstract

The aim of this systematic review is to determine whether Deep Learning (DL) algorithms can provide a clinically feasible alternative to classic algorithms for synthetic Computer Tomography (sCT). The following categories are presented in this study: ∙ MR-based treatment planning and synthetic CT generation techniques. ∙ Generation of synthetic CT images based on Cone Beam CT images. ∙ Low-dose CT to High-dose CT generation. ∙ Attenuation correction for PET images. To perform appropriate database searches, we reviewed journal articles published between January 2018 and June 2023. Current methodology, study strategies, and results with relevant clinical applications were analyzed as we outlined the state-of-the-art of deep learning based approaches to inter-modality and intra-modality image synthesis. This was accomplished by contrasting the provided methodologies with traditional research approaches. The key contributions of each category were highlighted, specific challenges were identified, and accomplishments were summarized. As a final step, the statistics of all the cited works from various aspects were analyzed, which revealed that DL-based sCTs have achieved considerable popularity, while also showing the potential of this technology. In order to assess the clinical readiness of the presented methods, we examined the current status of DL-based sCT generation.

## 1 Introduction

Image synthesis is an active area of research with broad applications in radiation oncology and radiotherapy (RT). This technology allows clinicians to bypass or replace imaging procedures if time, labor, or expense constraints prevent acquisition; there are certain circumstances when it is not advisable to use ionizing radiation; or there are instances when image registration can introduce unacceptable uncertainty between images of different imaging modalities. There have been many exciting clinical applications that have been developed as a result of these benefits, including the planning of RT with Magnetic Resonance Imaging (MRI) and the use of Positron Emission Tomography (PET)/MRI in tandem with RT treatment.

In recent decades, image synthesis has been investigated in relation to its potential applications. Traditionally, image conversion from one modality to another is carried out using models with explicit human-defined rules, which require adaptive parameter tuning on a case-by-case basis in order to achieve optimal results. Additionally, these models have varied characteristics based on the unique attributes of the imaging modalities involved, resulting in a variety of complex methodologies that are application-specific. In the case of anatomical imaging and functional imaging, it is particularly challenging to construct such models. It is for this reason that most of these studies employ Computed Tomography (CT synthesis from MRI) as the primary imaging tool.

It is now possible to combine image synthesis with other imaging modalities such as PET and Cone-Beam CT (CBCT) as a result of rapid advances in machine learning (ML) and computer vision over the last two decades (1). ML and Artificial Intelligence (AI) have been dominated for several years by deep learning (DL) as a broad sub-field within ML. To extract useful features from images, DL algorithms employ neural networks containing many layers and a large number of neurons.

Many networks have been proposed to achieve better performance in various applications. Data-driven approaches to image intensity mapping are commonly used by DL-based image synthesis methods. Generally, a network learns how to map the input to its target through a training stage, followed by a prediction stage where the target is synthesized from the input. In contrast to conventional model-based methods, a DL-based method can be generalized to multiple pairs of image modalities without requiring significant adjustments. By utilizing this approach, rapid translation to various imaging modalities is possible, allowing clinically relevant synthesis to be produced. Despite the effort required in collecting and curating data during network training, the prediction process usually takes only a few seconds. In medical imaging and RT, DL-based methods have demonstrated great promise because of these advantages.

In the domain of RT, MRI is preferred over CT for patient positioning and Organ at Risk (OAR) delineation (2–6) due to its better capacity to differentiate soft tissues (7). In RT conventionally, the primary imaging modality is CT. MRI is fused by deformable enrollment with CT scans because they deliver precise and high-resolution anatomy which is needed for dose calculations (2) for RT. However, residual mis-registration and variations in patient setup may introduce systematic errors that might influence the accuracy of the entire treatment. The point of MRI only RT is to eliminate the CT scans from the workflow and in its place use MR image(s) alone.

MRI-based treatments are getting very common because of the advancement of MR-guided treatment methods, e.g., MRI-linac (8). Here, online versatile RT utilizing MRI can be performed, exploiting the functional data and anatomy supplied by the modality (9) and reducing the registration error (2, 10, 11). Additionally, MR only RT can also help us protect the patient from the ionizing radiations and may decrease treatment cost (12) and workload (13).

Furthermore, similar techniques have been proposed to improve the quality of CBCT by converting a different imaging modality into sCT. Photon and proton therapy are effectively utilized using CBCT in image-guided adaptive radiotherapy (IGART). Despite this, the reconstruction of the image suffers from several artifacts such as shading, streaking, and cupping due to the severe scatter noise and truncated projections. As a result of these reasons, online adaptation of treatment plans does not commonly utilize daily CBCT. By converting CBCT to CT, it should be possible to compute accurate doses and provide patients with a better quality of treatment.

Furthermore, sCT estimation plays a significant role in PET attenuation correction (AC). The photon AC map from CT is often necessary for accurate PET quantification. A solution to the MRAC issue has been proposed to address this issue with the new hybrid PET/MRI scanners. The derivation of sCT from uncorrected PET can provide additional benefits to stand-alone PET scanners.

We present an in-depth review of emerging DL-based methods and applications for synthesising medical images and their applications in RT in this review. This review categorized recent literature according to their DL methods and highlighted their contributions. A survey of clinical applications is presented along with an assessment of relevant limitations and challenges. We conclude with a summary of recent trends and future directions.

## 2 Materials & methods

**Figure 1:**
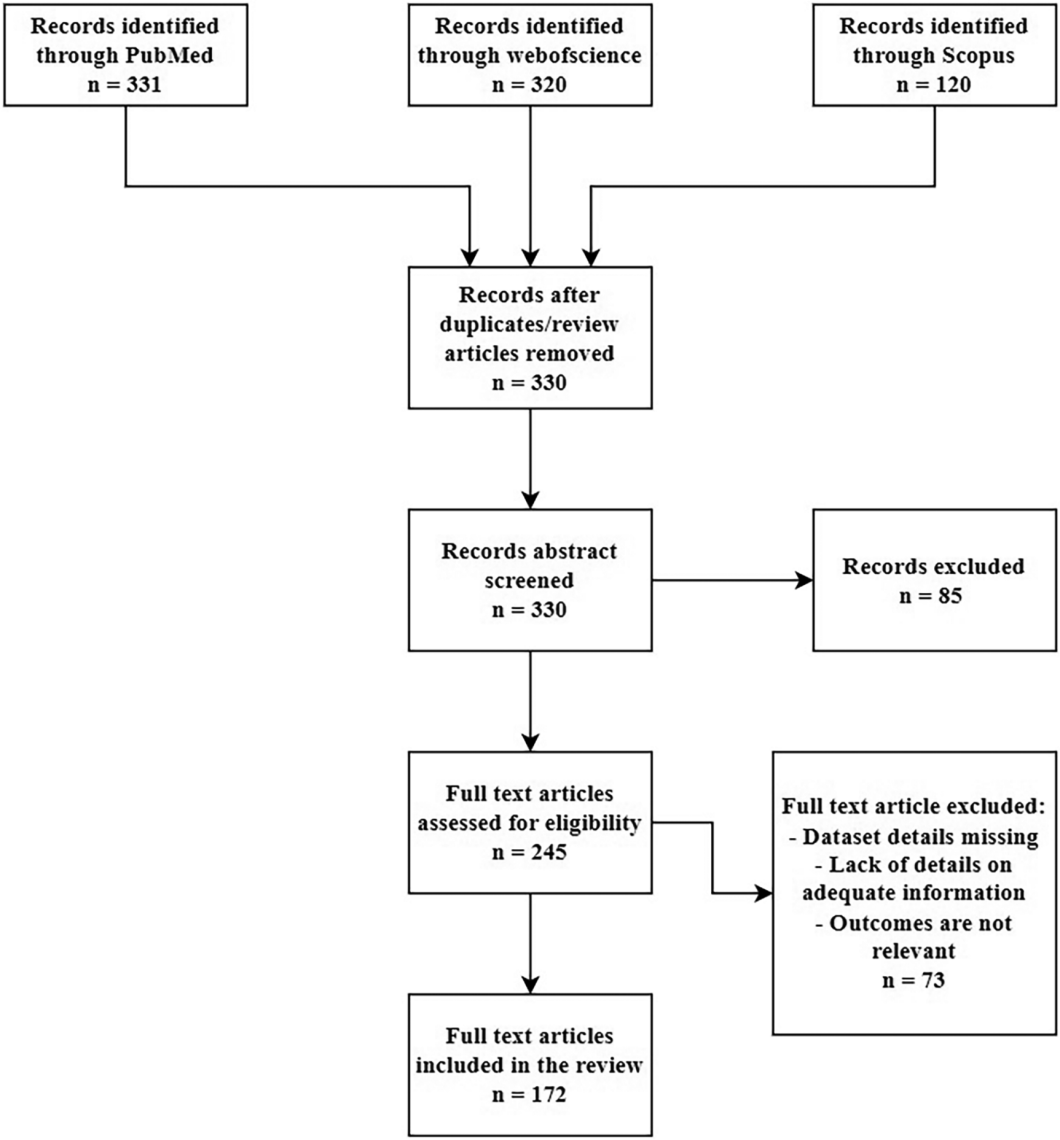
Article screening based on PRISMA guidelines.

We looked through the Scopus, PubMed and ScienceDirect electronic databases from January 2018 to Jun 2023 utilizing the associated keywords:((“radiotherapy” OR “radiation therapy” OR “MR-only radipotherapy” OR “proton therapy” OR “oncology” OR “imaging” OR “radiology” OR “healthcare” OR “CBCT” OR “cone-beam CT” OR “Low dose CT” OR “PET” OR “MRI” OR “attenuation correction” OR “attenuation map”) AND (“synthetic CT” OR “sCT” OR “pseudo CT” OR “pseudoCT” OR “CT substitute”) AND (“deep learning” OR “convolutional neural network” OR “CNN” OR “GAN” OR “Generative Adversarial Network” OR artificial intelligence)). We just selected original research papers written in English excluding the review papers. This review was conducted based on the PRISMA guidelines. The screening criteria is given in the Figure 1.

**Figure 2:**
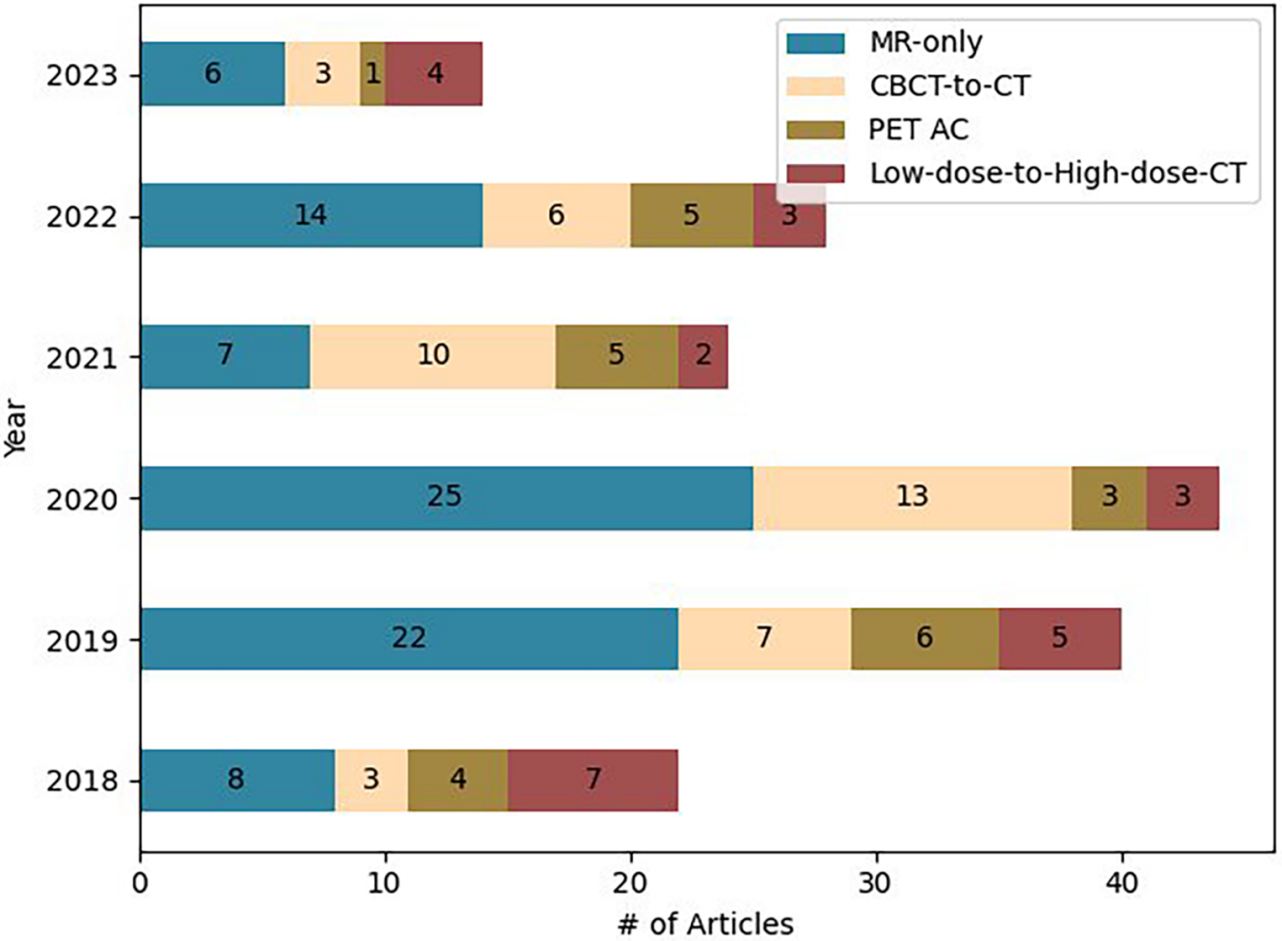
Number of articles selected for this study.

For each paper, we screened: Magnetic Resonance (MR) devices, MR images and sequences, number of patients, dataset split details (training, validation, and testing set), pre and post-processing of dataset, Deep learning (DL) technique utilized, loss functions, metrics used for the image comparison and dose evaluation. Figure 2 provides the information regarding the articles selected for this study.

### 2.1 Deep learning in medical images

**Figure 3:**
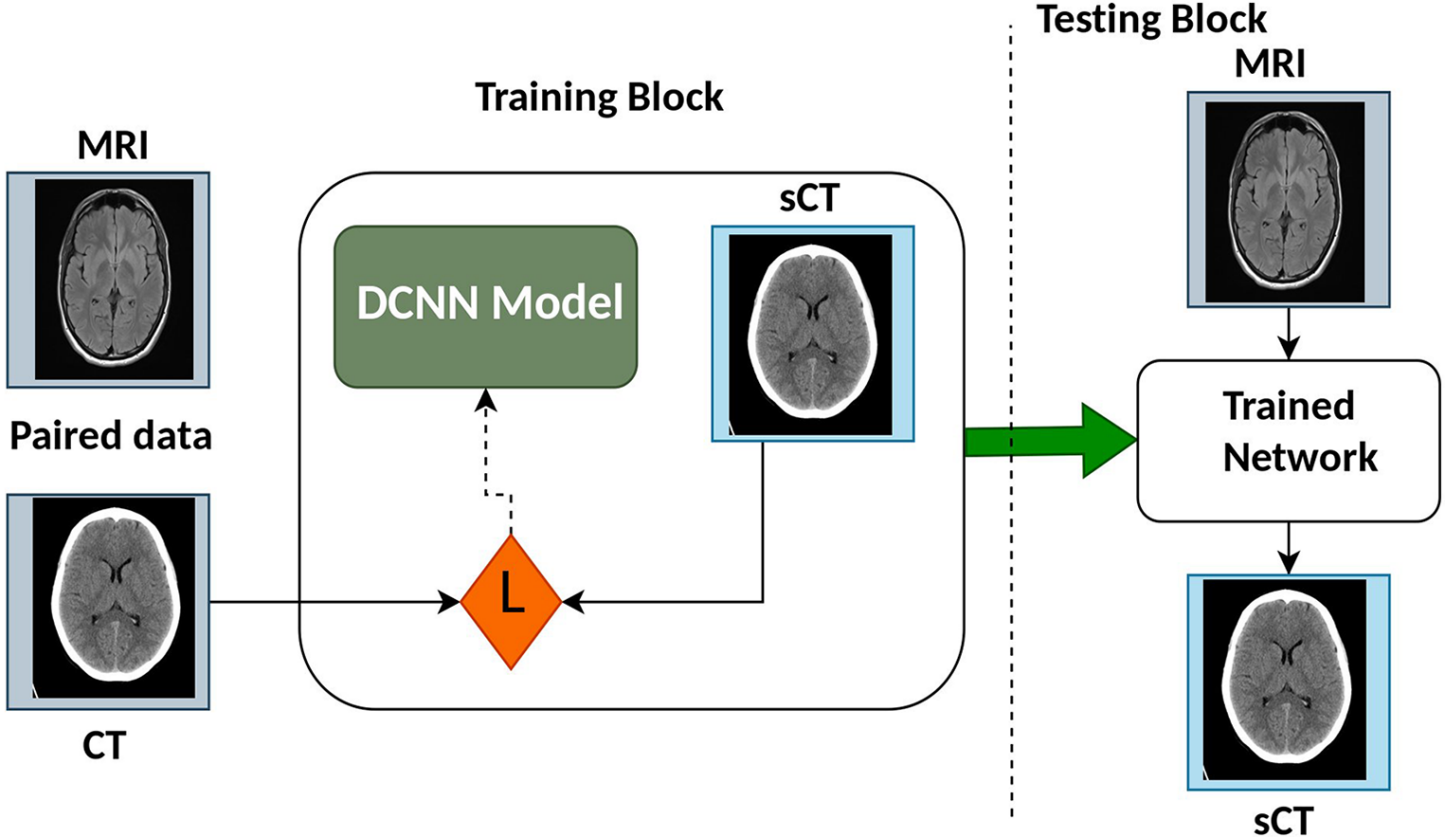
The architecture overview of convolutional neural network (CNN).

Deep Learning (DL) is a specialized subset of machine learning (ML) that focuses on deep neural networks and automated feature extraction. It has achieved remarkable success in tasks with large datasets, but it comes with higher computational requirements and challenges in interpretability compared to traditional ML methods. The choice between DL and ML depends on the specific problem, dataset size, and computational resources available. Recent reviews provide further insight into DL network architectures for medical imaging and RT (14–20). The synthetic Computed Tomography (sCT) generation using DL methods generally utilizes Convolution Neural Network (CNN)/Deep Convolution Neural Network (DCNN) or Generative Adversarial Network (GAN) and variants. Figure 3 shows the architecture of some CNN/DCNN and GAN networks.

#### 2.1.1 Convolution neural network (CNN)

Convolution Neural Network (CNN) is a famous class of deep neural networks utilizing a bunch of convolution filters for distinguishing image features. A CNN comprises an input layer, several hidden layers and an output layer.

CNN take an input image/feature vector (one information node for every passage) and change it through a progression of a series of hidden layers, regularly utilizing nonlinear activation functions. Each hidden layer is likewise comprised of a bunch of neurons, where every neuron is completely associated with all neurons in the previous layer. The last layer of a neural network (i.e., the “output layer”) is likewise completely associated and addresses the last result classification of the network. Several types of layers are utilized to build a CNN but the most common ones include:

• Convolutional (Conv) - These layers apply a convolution to the information, passing the outcome to the following layer. A convolution changes over every one of the pixels in its open field into a single value, resulting in a vector.
• Activation (ACT or RELU, where we use the same or the actual activation function) – The decision of activation function in the hidden layer will control how well the network model learns the training dataset. The decision of enactment work in the result layer will characterize the kind of predictions the model can make. Nonlinear activation functions (Rectified Linear Units (ReLU) (21), Leaky-RELU (22), Parametric-ReLU (PreLU) or exponential linear unit (ELU) (23)) play a crucial role in discriminative capabilities of the deep neural networks. The ReLU layer protects the information and is a commonly utilized activation layer because of its computational minimalism, authentic sparsity, and linearity.
• Pooling (POOL) - These layers are used to reduce the dimension (subsampling) of the feature maps. It decreases the number of parameters to learn and computation in the network. The pooling layer sums up the features present in a region of the feature map produced by a convolution layer. It stabilizes the learning process and also reduces the training epochs required.
• Fully connected (FC) – These layers are used to connect all the inputs from a layer with the activation function of the next layer.
• Batch normalization (BN) (24) - This layer permits each layer of the network to learn more freely. It is utilized to standardize the result of the previous layers.
• Dropout – This layer is used to prevent overfitting in the model. During each step of training time, it set the input units to 0 randomly.
• Softmax – This is the last layer in a neural network that performs multi-class characterization.

**Figure 4:**
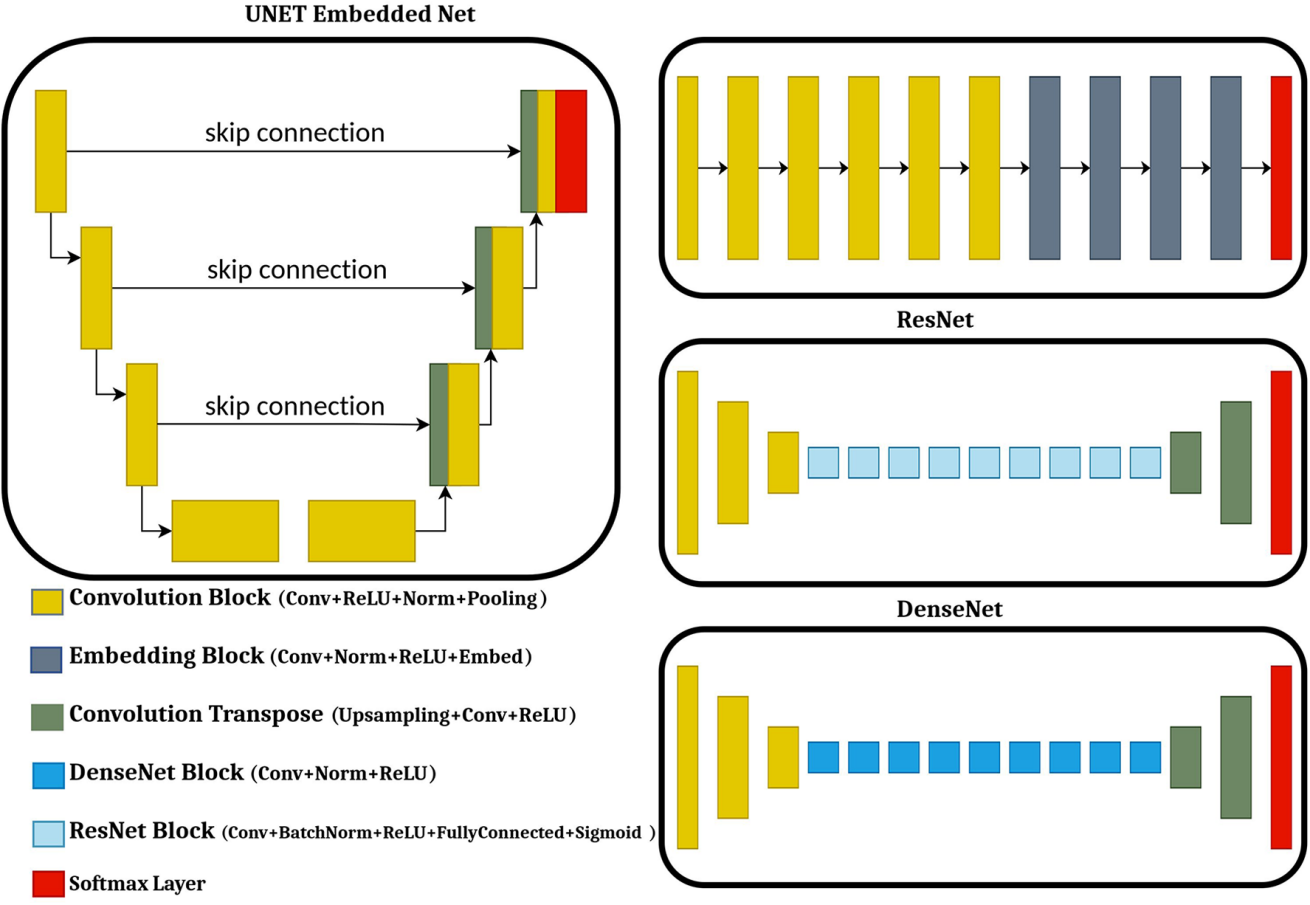
Different types of convolutional neural networks (CNNs).

During training stage, the model attempts to limit a true capacity called loss function, which is a intensity based similarity estimation between real image and the generated image. Figure 4 presents the architecture of CNN models commonly utilized for synthetic image generation. In the literature, the variations of CNN model incorporate convolution encoder-decoder (CED) (25), DCNN (26), Fully convolutional network (FCN) (27), U-Net (28–43), ResNet (44), SE-ResNet (45), and DenseNet (46).

The CNN network comprises combined encoder and decoder networks. CNN has been broadly utilized in DL literature due to its groundbreaking results (47–49). In the encoding part, it uses the method of downsampling to translate the low-level features map to a high-level features map. In the decoding part, the transposed convolution layer’s function is to translate the high-level feature maps to low-level feature maps to generate the synthetic image. The encoder part of the network utilizes a bunch of consolidated 2D convolution for distinguishing image features, followed by normalization, activation function and max pooling.

The decoder part utilizes transposed convolutional layers to join the feature and spatial information from the encoding part, followed by concatenation, up-sampling, and convolutional layers with a ReLU activation function.

The most notable and well-known CNN model is the U-shaped CNN (U-Net) architecture proposed by Ronneberger et al. (50). The U-Net architecture has direct skip connections between the encoder and decoder that helps in extracting and reconstructing the image features.

#### 2.1.2 Generative adversarial network (GAN)

**Figure 5:**
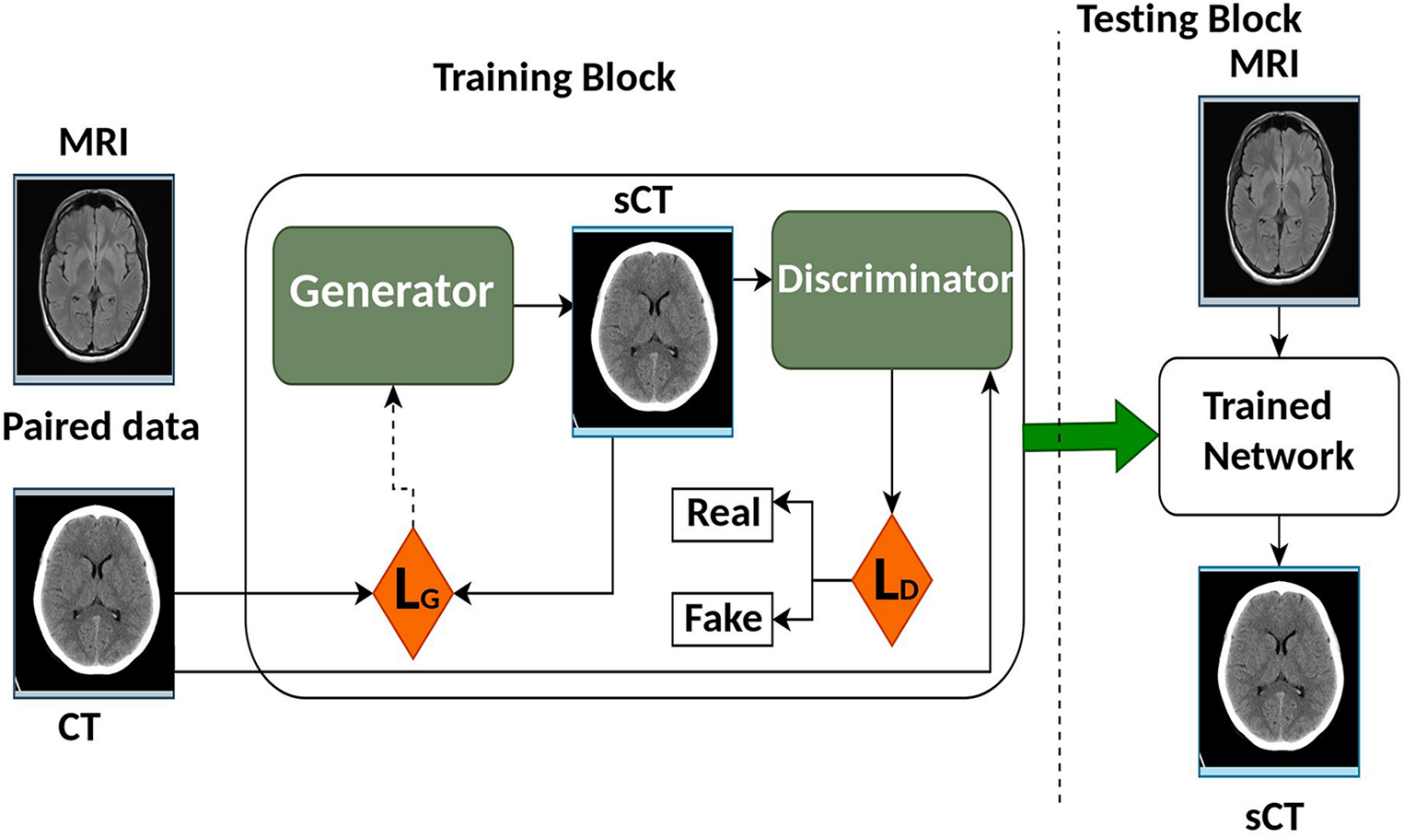
The architecture overview of generative adversarial network (GAN).

Generative adversarial network (GAN) was first introduced in 2014 by Goodfellow et al. (51). It improved the quality of image generation as compared to the previous Convolution Neural Network (CNN) models. The architecture of GAN as shown in Figure 5 trains two separate neural networks, the generator (G) and the discriminator (D). G attempts to create synthetic images while D on the other side decides if that image looks like the real image or not (52, 53). GAN presents an information-driven regularizer, it tries to improve itself and guarantees that the learned features bring the outcome close to the ground truth.

In basic GAN architectures, D and G are executed as Multi-Layered Perceptrons (MLPs). U-Net is the most used architecture as the G for GAN. Another frequently used G in GANs is the ResNet, as highlighted in the work by Emami et al. (54). ResNet stands out for its ease of optimization and its ability to reliably produce the desired results.

For the D part of the GAN, PatchGAN is used and it comprises six convolutional layers with different filters but the same kernel size and stride, trailed by five fully connected layers. For activation purposes, ReLU is utilized and for the convolution layer, batch normalization is utilized. The dropout layer is added to the fully connected layers, and in the last fully connected layer, a sigmoid function is utilized. The traditional GAN model uses adversarial loss ($L_{adv}$) as the cost function and it helps the network to produce better-looking sCT images with less blurry features (55, 56) compared to the images generated by other CNN models.

The discriminator attempts to boost it while the generator attempts to limit it as mentioned in the equation. $L_{adv}=E_{x}[logD(x)]+E_{z}[log(1-D(G(z))]$ Where, D(x) is the discriminator’s estimate if that real data instance x is real, $E_{x}$ and $E_{z}$ is the expected value over all real data and random instances respectively. G(z) is genertor’s output over noise (z) while D(G(z)) estimate if a fake instance is real. The formula derives from the cross-entropy between the real and generated distributions. The generator can’t directly affect the log(D(x)) term in the function, so, for the generator, minimizing the loss is equivalent to minimizing log(1 - D(G(z))) given a discriminator.

The most common variants of GAN used for synthetic image generation are Conditional GAN (cGAN) and cycle-GAN. The first cGAN architecture to generate synthetic Computed Tomography (sCT) to Magnetic Resonance Images (MRI) was proposed by Emami et al. (54). Unlike standard GAN, both the G and D of cGAN perceive the input image dataset. This approach tends to be more accurate as compared to previous approaches. Unlike standard GAN, several studies have been proposed to include SE-ResNet (41, 43), U-Net (44, 45, 57), DenseNet (46) and Embedded Net (26) as Generator for the cGANs. Evaluation of all four G, Embedded Net, DenseNet, SE ResNet and U-Net in cGAN is proposed by Fetty et al. (58) to generate synthetic images from MR T2 weighted images.

Several studies used a cGAN architecture to generate sCT from MRI (35, 41–43, 57, 59–69).

Cycle-GAN are commonly used to train Deep Convolutional Neural Networks (DCNN) to translate image-to-image. Cycle-GAN consists of two G and two D. In synthetic image generation using cycle-GAN, one G is used to generate sCT from MRI and the other to generate sMRI from sCT. A cyclic loss function is used to learn concurrently the features between the two modalities. The unpaired dataset is used to learn the mapping between two modalities and in some cases, it outperforms GANs using paired datasets (55).

#### 2.1.3 Loss functions used in deep learning models

Loss functions play an important role in guiding model training. Different loss functions are used based on the requirement and network configuration. L1 norm (70) and L2 (62, 69) are used frequently used to avoid overfitting and control complexity of model. L1 as compared to L2 is used more often due to its robustness to outliers in training data and it tends to perform better for image generation tasks. An image fidelity loss is commonly calculated by subtracting the average squared difference between the predicted and actual image, which is commonly referred to as Mean Square Error (MSE). It is imperative to maintain fine details when structural similarity index (SSIM) loss is applied while cross entropy loss is widely used for classification problem. Adversarial Loss is utilized by Generative Adversarial Networks to create realistic images. Some combination of different loss functions are also utilized in GANs and other models to improve the model accuracy (40, 41, 58, 64).

### 2.2 Dataset, dataset size & training dataset

The challenging part of the DL-based approaches applied on image synthesis is the paucity of datasets available for training and testing the different methods. Several studies are conducted with a minimum of 10 patients. Studies have also suggested that a higher number of images in the dataset can improve the performance of Deep Learning (DL) models. To improve model performance, diversity of training datasets is required. The images used for most of the studies were taken from adult patients. For training the model, most of the studies were conducted using paired datasets (where the images from both the modalities are given as input to learn the features) and very few studies used unpaired datasets. Some studies also compared the results on paired datasets over unpaired datasets (29, 60, 71, 72). Most commonly used networks were 2D networks, where 3D images were sliced into 2D images for training the network. Multiple configurations were also investigated in some studies (40, 73) described in this review. The most popular architecture for the image synthesis was Generative Adversarial Network (GAN), followed by U-Net and other Convolution Neural Networks (CNN). For the generator (G) of the GAN, mostly U-Net was used. Data augmentation is also used to train the network with different features and properties using small samples within the training dataset. Some conventional data augmentation techniques (19, 30, 63) such as rotation, translation, noise addition and deformations can be used with the training dataset. In this review, several training strategies were utilized: single-fold validation, k-fold cross-validation and leave-one-out validation. For single-fold cross-validation, the dataset is divided into two sets: one for training and the other for testing. For k-fold cross-validation, the dataset is separated into k number of subsets. For each training, one k subset is utilized for the testing phase and the remaining k subsets for the training phase. Leave-one-out validation is equivalent to k-fold validation with k being the number of samples in the training dataset.

### 2.3 Evaluation metrics

In the literature, several metrics are reported based on the image similarity or intensity, accuracy based on the geometry and evaluation of the dose for the radio therapy (RT). The metrics used in the literature are provided in Table 1. To evaluate the quality of the synthetic image based on voxels, the most used similarity metrics are Mean Absolute Error (MAE), Structural Similarity (SSIM), and Peak Signal to Noise Ratio (PSNR).

**Table 1:** Metrics reported in literature for synthetic image analysis using ground truth as reference.

	Type of Metric	Metrics	Ideal Value
Intensity Based Metrics	Mean Error	$ME=\frac{1}{N}sum_{i=1}^{N}sCT_{i}-CT_{i}$	0 HU
	Mean Absolute Error	$MAE=\frac{1}{N}sum_{i=1}^{N}|sCT_{i}-CT_{i}|$	0 HU
	Peak Signal to Noise Ratio	$PSNR=10log_{10}(\frac{Q^{2}}{MSE})$	Max of dB
	Structural Similarity Metric	$SSIM=\frac{\left(2\mu_{x}\mu_{y}+C_{1})(2\delta_{xy}+C_{2}\right)}{\left(\mu |x^{2}+\mu |y^{2}+C_{1})(\delta |x^{2}+\delta |y^{2}+C_{2}\right)}$	1
	Mean Square Error	$MSE=\frac{1}{N}\sum_{i=1}^{N}(sCT_{i}-CT_{i}{)}^{2}$	0
	Root Mean Square Error	$RMSE=\sqrt{\frac{1}{N}\sum_{i=1}^{N}(sCT_{i}-CT_{i}{)}^{2}}$	0 HU
	Normalized Cross Correlation	$NCC=\frac{1}{N}\sum_{x,y,z}\frac{\left(I_{CT}(x,y,z)-\mu_{CT})(I_{sCT}(x,y,z)-\mu_{sCT}\right)}{\delta_{CT}\delta_{sCT}}$	
Geometric Fidelity	Dice Similarity coefficient	$DSC=\frac{2(V_{CT}\cap V_{\;pCT})}{V_{CT}+V_{\;pCT}}$	1
	Hausdorff Distance	$H(sCT,CT_{ref})=max(h(sCT,CT_{ref}),h(CT_{ref},sCT))$	0 mm
	Mean Absolute Surface Distance	$MASD(A,R)=\frac{d_{ave}(S_{A},S_{R})+d_{ave}(S_{R},S_{A})}{2}$	
Dose difference metrics	Voxel-to-Voxel Dose Differences	Difference between the dose distribution computed on the reference CT and on the sCT	0 Gy or 0 %
	Dose Volume Histogram Difference	Dose differences on DVH specific points (Dmax, D70Gy, etc.), for a given structure	0 Gy or 0 %
Gamma Analysis	Mean Gamma	Value of the mean gamma	0
	Gamma pass-rate	Percentage of pixels/voxels with a gamma value lower than 1	100%

Besides voxel-based metrics, geometric accuracy can also be assessed by comparing delineated structures with corresponding voxel-based metrics. In terms of evaluating the accuracy of depicting specific tissue classes and structures, the Dice Similarity Coefficient (DSC) is a commonly used metric. DSC is calculated after applying morphological operations to binary masks and applying a threshold to Computed Tomography (CT) and synthetic CT (sCT). In addition to the Hausdorff distance, the mean absolute surface distance can be used to assess the segmentation accuracy, as it measures the distance between two contour sets (74).

A comparison of dose calculation between sCT and CT is generally performed using specific regions of interest (ROI) for both photon (f) and proton (p) RT. The most commonly used voxel-based metric dose difference (DD) is calculated by taking the average dose ($D_{CT}D_{sCT}$) of the ROI and redistributing it across the whole body, target, or other structures of interest. DD is expressed as a percentage of the prescribed dose (%) or the maximum dose (Gy), either relative to it or an absolute value. DD is directly correlated to the dose pass rate, which is the percentage of voxels with DD below a specified threshold.

Gamma analysis can be conducted in both 2D and 3D, offering a combined evaluation of dose and spatial factors. However, this process involves the configuration of multiple parameters, such as dose criteria, distance-to-agreement criteria, and dose thresholds. It’s important to note that there is no standardized approach for interpreting and comparing gamma index outcomes across various studies. The results can significantly differ due to variations in parameters, grid sizes, and voxel resolutions (75, 76). As a result, the gamma pass rate (GPR) is typically expressed as the percentage of voxels within a region of interest (ROI) that meet a specific threshold based on the reference dose distribution.

The dose-volume histogram (DVH) is a tool used routinely in clinical practice. As a general rule, clinically significant DVH points are reported in an evaluation of sCT. Also, range shift (RS) is considered in proton RT. In this case, the ideal range is determined as the distance from the distal dose fall-off ($R_{80}$) point at which the dose is at 80% of the maximum (77). As well as absolute RS error (RSe) expressed as the shift in the prescribed range relative to the actual beam direction ($RSe=R_{80CT}-R_{80sCT}$), relative RS error (%RS) can also be specified.

## 3 Results

### 3.1 MR to synthetic CT (sCT) generation for radiotherapy

A significant amount of research has been published in this field on the problem of Magnetic Resonance Image (MRI) to synthetic Computer Tomography (sCT) image synthesis as one of the first applications utilizing Deep learning (DL) for medical image analysis. The results for this section is provided in Table 2 CT acquisition is being replaced by MR-based CT synthesis primarily for clinical reasons (78). Despite recent improvements in sCT imaging, they are still inconclusive as diagnostic tools. The tool is also valuable for non-diagnostic settings, such as treatment planning and PET Attenuation Correction (AC).

**Table 2:** Overview of sCT generation methods for MR-only RT with image-based and dose difference evaluation.

Reference	Year	Tumor	Dataset	MRI	Deep Learning		Image Evaluation			Plan	Dose Evaluation			
					Arc	Config	MAE [HU]	PSNR [dB]	SSIM		DD [%]	GPR [%]	DVH	Others
Nie (56)	2018	Brain	16 TR	$T_{1w}$	GAN	2Dp	92.5 ± 13.9	27.6 ± 1.3						
Emami (54)	2018	Brain	15 TR, 5-x CV	$T_{1w}$	GAN	2Dp	89.3 ± 10.3	26.6 ± 1.2	0.83 ± 0.03					Tissues
Xian (26)	2018	Brain	16 TR, LOO	$T_{1w}$	AE	2.5Dp	85.4 ± 9.24	27.3 ± 1.1						
Dinkla (79)	2018	Brain	26 TR, 26 TE, 2-x CV	1.5T $T_{1w}$ GRE	CNN	2Dp	67 ± 11			f	−0.1 ± 0.3	99.8 ± 0.7		beam $\gamma_{3}$ depth $\gamma_{1}$, ME tissues
Xian (26)	2018	Prostate	22 TR, LOO	$T_{1w}$	AE	2.5Dp	42.4 ± 5.1	33.5 ± 0.8						
Maspero (64)	2018	Prostate	32 TR, 30 TE		GAN	2Dp	65 ± 10							
Chen (36)	2018	Prostate	36 TR, 15 TE	3T $T_{2w}$ TSE	U-Net	2Dp	30 ± 5			f	0.16 ± 0.09	99.4	< 0.2Gy	$\gamma_{3}$ $\gamma_{1}$, ME tissues
Arabi (32)	2018	Prostate	39 TR, 4-x CV	3T $T_{2w}$	U-Net	2Dp	33 ± 8			f	−0.01 ± 0.64	98.5 ± 0.7	< 3%	$\gamma_{3}$, $\gamma_{1}$, ME, DSC
Xu (80)	2019	Abdomen	10 TR, 10 TE, LOO	m-Dixon	GAN	2Dp	61 ± 3							
Lei (81)	2019	Brain	24 TR, LOO	$T_{1w}$	GAN	3Dp	55.7 ± 9.4	26.6 ± 2.3						
Jin (82)	2019	Brain	98 CT, 84 MR TR, 10 TE	3T $T_{2w}$	GAN	2Dp/u	19 ± 3	65.4 ± 0.9	0.25 ± 0.01					
Liu (25)	2019	Brain	40 TR, 10 TE	1.5T GRE post-Gd	CNN	2Dp	75 ± 23			f	< 0.2 ± 0.5	99.2		DSC
Kazemifar (43)	2019	Brain	54 TR, 9 VAL, 14 TE, 5-x CV	1.5T $T_{1w}$ post-Gd	GAN	2Dp	47 ± 11			f	−0.7 ± 0.5	99.2 ± 0.8	< 1	$\gamma_{3}$, $\gamma_{1}$
Shafai (83)	2019	Brain	25 TR, 2 VAL, 25 TE	1.5T $T_{1w}$ GRE	GAN	3Dp	55 ± 7			f	< 2	98.4 ± 3.5	< 1.65	ME, DSC, range, $\gamma_{3}$, $\gamma_{1}$
Gupta (33)	2019	Brain	47 TR, 13 TE, 5-x CV	3T $T_{1w}$	U-Net	2Dp	81 ± 15			f	2.3 ± 0.1			ME air, tissues
Neppl (40)	2019	Brain	55 TR, 28 VAL, 4 TE	1.5T $T_{1w}$ GRE	U-Net	2Dp	116 ± 26			f	> 982,98 ± 2			range, $\gamma_{1}$
					U-Net	3Dp	137 ± 32${\;}^{"}$			p	> 982,97 ± 3			ME
Spadea (28)	2019	Brain	12 TR, 2 VAL, 1 TE, LOO	3T $T_{1w}$ GRE	U-Net	2D+p	54 ± 7			p	0.00 ± 0.01			range, ME, DSC tissues
Koike (63)	2019	Brain	15 TR, 5-x CV	$T_{1w}$, $T_{2w}$ FLAIR	GAN	2Dpp	108 ± 24			f	0.7	99.2 ± 1.0	< 1	tissues, beam $\gamma_{3}$, depth $\gamma_{1}$
Olberg (41)	2019	Breast	12 TR, 18 v	1.5T GRE m-Dixon	GAN	2Dp	94 ± 11			p	< 0.5	98.4 ± 3.5		DRR, dis bone, NCC
							103 ± 15							
Jeon (84)	2019	Breast	14 TR, 2 TE, LOO	N/A	U-Net	2Dp								DSC: 74.76
Dinkla (34)	2019	H & N	22 TR, 12 TE	3T in-phase Dixon $T_{2w}$	U-Net	2Dp	75 ± 9			f	−0.07 ± 0.22	95.6 ± 2.9		$\gamma_{3}$, ME, DSC bone
Klages (69)	2019	H & N	15 TR, 12 TE	3T $T_{1w}$ GRE	GAN	2Dp		68 ± 2		p	< 0.5	< 98	< 0.5	SSIM, RMSE
Wang (31)	2019	H & N	23 TR, 10 TE	1.5T $T_{2w}$	U-Net	2Dp	131 ± 24							MAE, ME
Liu (85)	2019	Liver	21 TR, LOO	3T $T_{1w}$ GRE	GAN	3Dp	73 ± 18	22.7 ± 3.6		p		99.4 ± 1	< 1	range, $\gamma_{3}$, NCC
Fu (73)	2019	Prostate	20 TR, 5-x CV	1.5T $T_{1w}$ w/o C	U-Net	2Dp	40.5 ± 5.4							
						3Dp	37.6 ± 5.1							
Liu (85)	2019	Prostate	17 TR, LOO	1.5T $T_{2w}$	GAN	3Dp	51.32 ± 16.91							
Lei (81)	2019	Prostate	20 TR, LOO	3T $T_{2w}$	GAN	3Dp	51 ± 16	24.5 ± 2.6						
Florkow (38)	2019	Prostate	27 TR, LOO	3T in-phase Dixon $T_{1w}$	U-Net	3Dp	32 ± 8	36.5 ± 1.6						MAE, DSC
Liu (86)	2019	Prostate	17 TR, LOO	1.5T $T_{2w}$	GAN	3Du	51 ± 17	24.2 ± 2.5		p	−0.07 ± 0.07	98 ± 6	< 1	NCC, $\gamma_{3}$, $\gamma_{1}$
Largent (87)	2019	Prostate	25 TR, 14 TE, 3-x CV	3T $T_{2w}$ TSE	U-Net	2Dp	34 ± 8			f	< 1	99.2 ± 1	< 1	tissues, ME
					GAN	2Dp	34 ± 8				< 1	99.1 ± 1		
Fu (60)	2020	Abdomen	12 TR, 4-x CV	0.3T GRE	GAN	2Dp	90 ± 19	27.4 ± 1.6		f+$B_{0}$	< ± 0.6	98.7 ± 1.5	< ± 0.15	$\gamma_{3}$
				1.5T GRE		2Du	94 ± 30	27.2 ± 2.2			< ± 0.6	98.5 ± 1.6		
Liu (88)	2020	Abdomen	46 TR, 31 TE, 3-x CV	3T $T_{1w}$ GRE	U-Net	2.5Dp	79 ± 18			f			< 2Gy	MAE, ME organs
Cusumano (59)	2020	Abdomen	39 TR, 19 TE	0.35T GRE	U-Net	2Dp	79 ± 18			f+$B_{0}$	< 0.1	98.7 ± 1.1	< 2.5	ME, $\gamma_{3}$, $\gamma_{1}$
Florkow (89)	2020	Abdomen	54 TR, 18 VAL, 12 TE, 3-x CV	1.5T $T_{1w}$	U-Net	3Dp	62 ± 13	30.0 ± 1.8		f, p	< 0.1	99.7 ± 0.3	< 2	beam depth
				3T GRE $T_{2w}$ TSE	U-Net	3Dp					< 0.5	96.2 ± 4.0	< 3	ME, DSC tissues
Koike (63)	2020	Brain	15 TR	$T_{1w}$	GAN	2Dp	108.1 ± 24.0							
Yang (90)	2020	Brain	28 TR, 2 VAL, 15 TE	1.5T $T_{1w}$	GAN	2Dp	134 ± 12	24.0 ± 0.9	0.76 ± 0.02					
Xu (72)	2020	Brain	33 TR, LOO	$T_{1w}$	GAN	2Du	9.0 ± 0.8		0.75 ± 0.77					
					U-Net	2Dp	65 ± 4	28.8 ± 0.6	0.972 ± 0.004					
Maspero (91)	2020	Brain	30 TR, 10 VAL, 20 TE, 3-x CV	1.5T,3T $T_{1w}$ GRE post-Gd	GAN	2Dp	61 ± 14	26.7 ± 1.9	ME, DSC, SSIM	f, p	0.1 ± 0.4, −0.1 ± 0.3	99.5 ± 0.8, 99.6 ± 1.1	< 1%, < 3	beam, depth $\gamma_{3}$
Kazemifar (57)	2020	Brain	66 TR, 11 TE, 5-x CV	1.5 $T_{1w}$ post-Gd	GAN	2Du	78 ± 11			p	0.3 ± 0.3	99.2 ± 1.02	< 3	beam $\gamma_{3}$, depth $\gamma_{1}$
Massa (92)	2020	Brain	81 TR, 11 TE, 8-x CV	1.5T 3D T1 GRE	U-Net	2Dp	45.4 ± 8.5	43.0 ± 2.0	0.65 ± 0.05					
				1.5T 3D T1 GRE Gd	U-Net		44.6 ± 7.4	43.4 ± 1.2	0.63 ± 0.03					
				1.5T 2D T2 SE	U-Net		45.7 ± 8.8	43.4 ± 1.2	0.64 ± 0.03					
				1.5T 2D T2 Flair	U-Net		51.2 ± 4.5	44.9 ± 1.2	0.61 ± 0.04					
Li (71)	2020	Brain	28 TR, 6 TE	1.5T $T_{2w}$	GAN	2Du	94 ± 6	26.3 ± 0.6	0.955 ± 0.007					
Andres (29)	2020	Brain	242 TR, 81 VAL, 79 TE,	3T $T_{1w}$	CNN	3Dp	81 ± 22		tissues	f	0.13 ± 0.13	99.6 ± 0.3	< ± 0.15	$\gamma_{3}$, tissues
				1.5T GRE post-Gd	U-Net	3Dp	90 ± 21				0.31 ± 0.18	99.4 ± 0.5		
Tie (65)	2020	H & N	28 TR, 4 VAL, 8-x CV	1.5T PG $T_{1w}$	GAN	2Dp	76 ± 15	29.1 ± 1.6	0.92 ± 0.02					DSC
Kearney (93)	2020	H & N	60 TR, 30 TE	3T in-phase Dixon $T_{1w}$	GAN	2Du	19.6 ± 0.7	62.4 ± 0.5	0.78 ± 0.2					
Largent (53)	2020	H & N	7 TR, 8 TE, LOO	1.5T $T_{1w}$	GAN	2Dp	83 ± 49							
Qian (94)	2020	H & N	10 TR, LOO	1.5T $T_{1w}$	GAN	2Dp	42 ± 62							RMSE
Su (95)	2020	H & N	32 TR, 8 TE, 5-x CV	3T UTE	U-Net	2Dp	104 ± 21							DSC
Qi (35)	2020	H & N	30 TR, 15 TE	$T_{1w}$ + $T_{2w}$+ CE $T_{1w}$ + CE $T_{1w}$	GAN	2Dp	69.98 ± 12.02							
				3T $T_{1w}$ post-Gd	GAN	2Dp	70 ± 12	29.4 ± 1.3		p	−0.3 ± 0.2	97.8 ± 0.9		SSIM, DSC, DRR
				3T $T_{2w}$ TSE	U-Net	2Dp	71 ± 12	29.2 ± 1.3			−0.2 ± 0.2	97.6 ± 1.3		
Peng (68)	2020	H & N	135 TR, 10 VAL, 28 TE	3T $T_{1w}$ GRE	GAN	2Dp	70 ± 9		ME, DSC tissues	f	−0.1 ± 0.3	98.7 ± 1.0	< 1.5	beam depth, ME, DSC tissues
	2020				GAN	2Du	101 ± 8				0.1 ± 0.4	98.5 ± 1.1	< 1.5	
Thummerer (96)	2020	H & N	27 TR, 3-x CV	3T $T_{1w}$ GRE	GAN	2Dp	65 ± 4		ME, DSC	p	< ± 0.2	93.5 ± 3.4	< 1.5	NTCP, RS $\gamma_{3}$
Boni (62)	2020	Prostate	11 TR, 8 TE	1.5T and 3T $T_{2w}$	GAN	2Dp	48.5 ± 6							
Boni (62)	2020	Pelvis	11 TR, 8 TE	3T $T_{2w}$ TSE, 1.5T $T_{2w}$ TSE	GAN	2Dp	49 ± 6		ME organs	f	0.7 ± 0.4	99.2 ± 1.0	< 1.5	
Fetty (58)	2020	Pelvis	26 TR, 15 VAL, 10 TE	0.35T $T_{2w}$, 1.5T/3T $T_{2w}$	GAN	2.5Dp	41 ± 4	31.4 ± 1	ME, MSE bone	f	< ± 1		< 1.5	
Cusumano (59)	2020	Pelvis	39 TR, 14 TE	0.35T GRE $T_{2w}$	U-Net	2Dp	54 ± 12		tissues	f	< 0.5	99.0 ± 0.7	< 1	$\gamma_{3}$ $\gamma_{1}$
Bird (67)	2020	Rectum	46 TR, 44 TE	1.5T GRE $T_{2w}$	GAN	2Dp	35 ± 7		ME bone	f	< ± 0.8	99.8 ± 0.1	< 1	$\gamma_{3}$ $\gamma_{1}$
Ranjan (97)	2021	Brain	307 TR, 60 TE	3T $T_{2w}$	GAN	2Dp	0.030±0.017	21.422±3.964	0.823±0.063					MSE
Li (98)	2021	Brain	60 TR, 11 TE	T1/T2/Flair	GAN	2Dp	74.56 ± 8.61	28.30 ± 0.83	0.84 ± 0.01					RMSE
Tang (66)	2021	Brain	27 TR, 10 TE	3T $T_{1w}$	GAN	2Dp	60.52 ± 13.32					99.76		$\gamma_{3}$ $\gamma_{2}$
Touati (99)	2021	H & N	56 patients		GAN	2Dp	26.44 ± 0.62		0.74 ± 0.05					ME
Farjam (100)	2021	Prostate	20 TR, 10 TE, 3-x CV	0.35T $T_{1w}$	U-Net	3Dp	29.68 ± 4.41	31.06 ± 4.13						
Brou (101)	2021	Pelvis	19 TR, 19 TE	1.5T, 3T $T_{2w}$	GAN	2Du	59.8				1.2	> 99		$\gamma_{3}$
Zimmermann (102)	2021	Prostate	12 TR	0.35T $T_{2w}$	GAN	2Dp	44 ± 3			f, p	< 2			$\gamma_{3}$ $\gamma_{2}$
Szalkowski (103)	2021	Pelvis	11 patients	$T_{2w}$	CNN	3Dp	72.9 ± 88.1	31.2 ± 2.2				97.7 ± 0.7		$\gamma_{3}$ $\gamma_{2}$
				$T_{2w}$			71.1 ± 3.1							
				T1CM			82.9 ± 6.1							
Xu (80)	2022	Abdomen	10 TR, 10 TE, LOO	m-Dixon	GAN	2Dp	61 ± 3							
Li (104)	2022	Abdomen	37, 10-x CV	0.35T $T_{1w}$	U-Net	2Dp	35.64	24.11						NCC
Wang (105)	2022	Brain	150 TR, 20 VAL, 45 TE, 5-x CV	1.5T/3T T1/T2/Flair	GAN	2Dp	42 - 46			p				
jabbarpour (106)	2022	Brain	125 TR, 25 VAL, 39 TE	$T_{1w}$/$T_{2w}$	GAN	2Du	61.87 ± 22.58	27.05 ± 2.25	0.84 ± 0.05	3DCRT		98.96 ± 1.1	< 2	$\gamma_{3}$, $\gamma_{2}$
Zimmermann (107)	2022	Brain	33 TR, 6 VAL, 8 TE	$T_{1w}$	CNN	2Dp	79.8 ± 5.9			p	< 1			
Islam (108)	2022	Brain	12 TR, 2 VAL, 2 TE, 5-x CV	T1/T2/Flair	U-Net	3Dp		36.9242 ± 0.351	0.9827 ± 0.009					MSE
Wang (109)	2022	Brain	125 tr/v, 7 TE	1.5T, 3T $T_{1w}$	GAN	3Dp	65.3 ± 13.9	28.5 ± 2.2		p	0.3 ± 0.2	97.5 ± 1.1		$\gamma_{3}$, $\gamma_{2}$, SSIM
Zimmermann (110)	2022	Head	33 TR, 6 VAL, 8 TE	$T_{1w}$	U-Net	3Dp	79.8 ± 5.9		0.97 ± 0.01	p	< 1			PTV
					$T_{2w}$	3Dp	71.1 ± 3.1		0.98 ± 0.00					
					T1 CE	3Dp	82.9 ± 6.1		0.96 ± 0.01					
Chen (111)	2022	H & N	162 TR, 14 VAL, 30 TE	3T $T_{1w}$	GAN	2Dp	64.89 ± 5.31			p	0.58 ± 1.61	< 97.32	> 2 gy	
					GAN	3Dp	64.31 ± 4.61			p	0.47 ± 0.94	< 97.32	> 2 gy	
Scholey (112)	2022	H & N	96 TR, 6 VAL, 18 TE	1.5T $T_{1w}$	U-Net	3Dp	93.3 ± 27.5	0.97 ± 0.01			< 2	96.8 ± 2.6	98.9 ± 1.0	$\gamma_{3}$, $\gamma_{2}$
Vajpayee (113)	2022	Pelvic	11 TR, 8 TE	$T_{1w}$/$T_{2w}$	GAN	2Dp	40.4 ± 4.7	57.2 ± 1.4						ME
Tahri (114)	2022	Prostate	25 TR, 3 VAL, 11 TE	3T $T_{2w}$	GAN	2Dp	29.5 ± 7.9				≤ 1	99.4	≤ 0.2	$\gamma_{1}$
hsu (115)	2022	Prostate	15 TR, 2 VAL, 3 TE, loo	0.35T	GAN	3Dp	30.1 ± 4.2	35.2 ± 1.7	0.97 ± 0.0035			99		$\gamma_{1}$, $\gamma_{2}$
Lenkowicz (116)	2022	Thorax	32 TR, 8 VAL, 20 TE	0.35T $T_{1w}$	GAN	3Dp	54.9 ± 10.5			p	< 1	95.5 ± 5.9	< 5, 1 Gy	PTV, ME, $\gamma_{3}$, $\gamma_{2}$
Rippke (117)	2023	Abdomen	37, 10-x CV	0.35T $T_{1w}$	U-Net	2Dp	35.64	24.11						NCC
Hernandes (118)	2023	Abdomen	57 tr/VAL, 19 TE, 5-x CV	0.35T $T_{1w}$	U-Net	2Dp	28 ± 14.7				< 1	91, 95	0.1 ± 0.28	ME, $\gamma_{2}$, $\gamma_{3}$
					GAN	2Dp	25.9 ± 13.4							
Zhao (119)	2023	Brain	94 tr/VAL, 10 TE, 5-x CV	$T_{1w}$	U-Net	2Dp	67.81 ± 24.31				< 1	> 98	0.98 ± 0.01	$\gamma_{2}$
Zhao (120)	2023	H & N	64 tr/VAL, 15 TE	1.5T $T_{1w}$	GAN	2Dp	91.3 ± 10.9	27.4 ± 1.0	0.94 ± 0.01					DSC, MSD
Zhou (121)	2023	H & N	35 TR, 5 VAL, 10 TE	3T T1/T1c/		2Du	62.98 ± 5.28	28.51 ± 0.74	0.96 ± 0.01					NRMSE
				T2/T1 dixon										
Wyatt (122)	2023	Pelvis	36 tr/VAL, 20 TE	T1/$T_{2w}$ ZTE	U-Net	2Dp	36 ± 3				< 0.5	98.0 ± 0.4		$\gamma_{1}$, PTV

Abbreviations used in the following tables: AE → Auto-Encoder, Arc → Architecture, x CV → fold cross validation, CNN → Convolutional Neural Network, Config → Configuration, DSC → Dice Similarity Co-efficient, DRR → Digitally Reconstructed Radiograph, f → photon plan, FLAIR → Fluid Attenuated Inversion, GAN → Generative Adversarial Network, Gd → Gadolinium, GRE → Gradient Recalled Echo, H&N → Head and Neck, LOO → Leave One Out, m-Dixon → multi-contrast Dixon, MAE → Mean Absolute Error, ME → Mean Error, NCC → Normalized Cross Correlation, p → proton plan, PCC → Pearson Correlation Coefficient, PSNR → Peak Signal-to-Noise Ratio, RMSE → Root Mean Squared Error, SE → Spin Echo, SSIM → Structural Similarity, TR → Training, TE → Testing, TSE → Turbo Spin Echo, VAL → Validation, $\gamma_{1}$ → $\gamma_{1\%,1mm}$, $\gamma_{2}$ → $\gamma_{2\%,2mm}$, $\gamma_{3}$ → $\gamma_{3\%,3mm}$.

Radio therapy (RT) workflows commonly utilize MR and CT imaging for treatment planning on many patients. CT images provide electron density maps for dose calculation and reference images to position the patient prior to treatment. MR images offer excellent tissue contrast to diagnose gross tumours and organs at risk (OARs). Using image registration, treatment planning is performed by propagating MR contours to CT images. In addition to time and expense costs for the patient, combining both modalities contributes to systematic image fusion errors. Furthermore, CT may also expose patients to non-negligible doses of ionizing radiation (123), especially those requiring re-simulation. MRI-based treatment planning workflows would therefore be highly desirable instead of CT scans. Additionally, there is a growing demand for MRI exclusively for RT as MR linear accelerator (MR-linac) technology emerges.

Due to the lack of a one-to-one relationship between MR voxel intensity and CT’s Hounsfield Unit (HU is a quantitative measure to represent the radio density of tissues, helping in the differentiation of structures based on their properties), intensity-based calibration methods fail to deliver accurate and consistent results. CT imaging differs from MRI because in CT, air is dark and bone is bright. While translating MR images to CT, MR images are typically segmented into several classes of materials (e.g., air, soft tissue, bone) and then assigned CT HU values (11, 124–128) or registered to an atlas with known CT HU values (129–131). Segmentation and registration are the main components of both of these methods, which introduce significant errors due to ambiguous boundaries between bone and air, for instance, and significant inter-patient variations.

In literature, nearly all studies reported the image quality of their sCT using mean absolute error (MAE), peak signal-to-noise ratio (PSNR) and structural similarity (SSIM) metrics for CT synthesis applications in RT. Many studies also calculated the dose from the original treatment plan. Approximately 1% of the dose was different, which is small compared to the uncertainty associated with the total dose over the entire treatment course (5%).

In RT, DL-based methods generate relatively minor improvements in dosimetric accuracy compared to image accuracy and may not be clinically relevant. VMAT (Volumetric Modulated Arc Therapy) plans offer greater flexibility in dose calculations, particularly when dealing with image inaccuracies, especially in uniform areas like the brain. In VMAT, random image inaccuracies tend to balance out within an arc, but it’s worth noting that there’s a non-linear relationship between random image inaccuracies and dosimetric errors.

According to Liu et al. (85), most of the dose difference caused by sCTs occurs at the distal end of the proton beam due to errors along the beam path on the planning CT. As a result, the tumour could be substantially underdosed or Organs at risk overdosed. According to Liu et al. (85, 86), the largest absolute difference observed among patients with liver cancer is 0.56 cm, while for those with prostate cancer, the mean absolute difference is 0.75 cm. Besides assessing dosimetric accuracy for treatment planning, geometric fidelity is another essential consideration. Despite this, there are very few studies assessing sCT positioning accuracy. It has also been investigated whether sCT can work in proton therapy for prostate (86), liver (85), and brain cancers (83).

### 3.2 CBCT to synthetic CT (sCT) generation for radiotherapy

Synthetic Computed Tomography (sCT) using Cone beam CT (CBCT) is a physics problem that is governed by the same principles of x-ray attenuation and back projection. However, their application in clinical practice differs. So, we consider them as two distinct imaging modalities in this review. By comparing anatomic landmark displacements from the treatment planning CT images and CBCT images, image-guided radio therapy (IGRT) is used to check for patient setup errors and interfraction motion (132). More demanding applications of CBCT have been proposed with increased adoption of adaptive RT techniques, such as daily dose estimation and auto-contouring based on deformable image registration obtained through simulation with CT images (133, 134). The results for this section is provided in Table 3.

**Table 3:** Overview of sCT generation methods for RT with CBCT image-based and dose difference evaluation.

Reference	Year	Tumor	Dataset	Deep Learning		Image Evaluation			Plan	Dose Evaluation				
				Config	Arc	MAE [HU]	PSNR [dB]	SSIM		DD [%]	DPR [%]	GPR [%]	DVH	Others
Xie (135)	2018	Lung	15 TR, 5 TE	2Dp	AE		8.823							
Hansen (136)	2018	Prostate	15 TR, 8 VAL, 7 TE	2Dp	U-Net	46								Passing rate for 2% DD: 100% for x, 15%–81% for p
Kida (137)	2018	Prostate	16 TR, 4 TE, 5-x CV	2Dp	U-Net		50.9	0.967	No					SNU, RMSE
Harms (138)	2019	Brain	24 TR, LOO	3Dp	GAN	13 ± 2	37.5 ± 2.3		No					NCC, SNU
Chen (139)	2019	H & N	30 TR, 7 VAL, 7 TE	2Dp	U-Net	18.98	33.26	0.8911	No					RMSE tissues
Li (140)	2019	H & N	50 TR, 10 VAL, 10 TE	2Dp	U-Net	Jun-27			f	0.2 ± 0.1		95.5 ± 1.6	< 1	ME organs
Liang (141)	2019	H & N	81 TR, 9 VAL, 20 TE	2Du	GAN	29.9 ± 4.9	30.7 ± 1.4	.85 ± .03	f			98.4 ± 1.7, 96.3 ± 3.6		RMSE phantom
Harms (138)	2019	Prostate	20 TR	3Dp	GAN	16 ± 5	30.7 ± 3.7							NCC, SNU
Kurz (142)	2019	Prostate	18 TR, 8 TE, 4-x CV	2Du	GAN	87 ± 5			f, p		99.9 ± 0.3, 80.5 ± 5	95.9 ± 2.0	< ± 1.5%, < 1	$DPR^{1}$ $\gamma_{3}$, $DPR^{3}$ RS, ME
Landry (143)	2019	Prostate	27 TR, 7 VAL, 8 TE	2Dp	U-Net	58			f, p		> 98.41, 88.5	99.52, > 96.5		$\gamma_{1}$ $\gamma_{3}$ $DPR_{2}$, $DPR_{2}$ RS, ME
Maspero (144)	2020	Breast	15 TR, 8 VAL, 10 TE	2Du	GAN	66 ± 18	29.0 ± 2.1	.76 ± .02		0.1 ± 0.4		92 ± 8		
Barateau (145)	2020	H & N	30 TR, 14 TE	2Dp	GAN	82.4 ± 10.6			f	91.0 ± 5.32			< 1Gy, < 1	ME, tissues
Eckl (146)	2020	H & N	25 TR, 15 TE	2Dp	GAN	77.2 ± 16.6			f		91.5 ± 4.3	95.0 ± 2.4	< 2.4	$\gamma_{3}$, ME, DSC
Maspero (144)	2020	H & N	15 TR, 8 VAL, 10 TE	2Du	GAN	53 ± 12	30.5 ± 2.2	.81 ± .04		0.1 ± 0.5		97.8 ± 1		
Thummerer (96)	2020	H & N	22 TR, 11 TE, 3-x CV	2Dp	U-Net	36 ± 6			p	-0.1 ± 0.3		98.1 ± 1.2		RS, $\gamma_{3}$, ME, DSC, SNU
Yuan (147)	2020	H & N	50t TR, 10 TE	2.5Dp	U-Net	49.28	14.25	0.85	No					SNR
Zhang (148)	2020	H & N	135 TR, 15 VAL, 10 TE, 10-x CV	2.5Dp	GAN	24 ± 4	22.8 ± 3.4		p					RS
Maspero (144)	2020	Lung	15 TR, 8 VAL, 10 TE	2Du	GAN	83 ± 10	28.5 ± 1.6	.78 ± .04	f	0.2 ± 0.9		94.9 ± 3	< 2	$\gamma_{3}$, ME
Liu (149)	2020	Pancreas	30 TR, LOO	3Dp	GAN	56.9 ± 13.8	28.8 ± 2.5	.71 ± .03	f				< 1Gy	NCC, SNU
Eckl (146)	2020	Pelvis	205 TR, 15 TE	2Dp	GAN	42 ± 5			f		88.9 ± 9.3	98.5 ± 1.7	< 1	$\gamma_{3}$, ME, DSC, HD tissue
Kida (150)	2020	Pelvis	16 TR, 4 TE	3Du	GAN	0.688								
Eckl (146)	2020	Thorax	53 TR, 15 TE	2Dp	GAN	94 ± 32			f		76.7 ± 17.3	93.8 ± 5.9	< 2.6	$\gamma_{3}$, ME, DSC, HD tis
Zhang (148)	2020	Pelvis	135 TR, 15 VAL, 15 TE, 10-x CV	2.5Dp	2.5D GAN	8.1 ± 1.3	24 ± 7.5		f	< 1				
					2D GAN	8.1 ± 1.4	23.8 ± 1.8							
					CycleGAN	8.9 ± 3.1	22.1 ± 5.5							
					U-Net	19.2 ± 6.4	18.9 ± 6.7							
Dai (151)	2021	Breast	52 TR, 23 TE	3Dp	CNN	71.58 ± 8.78	23.34 ± 3.63	0.92 ± 0.02				91.46 ± 4.63		$\gamma_{3}$, ME, SNU
Gao (152)	2021	Chest	136 TR, 34 TE	2Dp	GAN	43.5 ± 6.69	29.5 ± 2.36	93.7 ± 3.88	p			91.4 ± 3.26		$\gamma_{1}$ $\gamma_{3}$
Xue (153)	2021	H & N	169 tr/v, 34 TE	2Dp	U-Net	26.8 ± 10.0	29.1 ± 1.7	0.94 ± 0.01				> 95		$\gamma_{1}$ $\gamma_{3}$, RMSE
					GAN	24.3 ± 8.0	31.3 ± 1.9	0.95 ± 0.01						
					CycleGAN	23.8 ± 8.6	37.8 ± 2.1	0.96 ± 0.01						
Chen (154)	2021	H & N	99 TR, 15 VAL, 29 TE	2Dp	CNN	28.52 ± 16.71	30.75 ± 3.89	0.9642 ± 0.0186						RMSE
Dahiya (155)	2021	Lungs	60 TR, 20 VAL, 15 TE	3Dp	GAN	29.31 ± 12.64	34.69 ± 2.41	0.92 ± 0.01						DSC
Liu (156)	2021	Lungs	32 TR, 8 VAL, 12 TR	2Dp	GAN	32.70 ± 7.26	34.12 ± 1.32	0.86 ± 0.04						RMSE
Thummerer (157)	2021	Lungs	22 TR, 11 TE, 3-x CV	2Dp	CNN	31 ± 4	30.7 ± 3.3	0.938 ± 0.019	p	± 0.5		96.8		$\gamma_{3}$, ME
Wu (158)	2021	Prostate	120 TR, 33 TE, 5-x CV		CNN	52.18								
Zhao (159)	2021	Rectum	30 TR, 10 TE	2Dp	GAN	52.99 ± 12.09	0.81 ± 0.03							DSC
Qiu (160)	2021	Thorax	20 patients, 5-x CV	3Dp	GAN	66.2 ± 8.2	30.3 ± 6.1	0.91 ± 0.05						NCC
Yuan (161)	2022	H&N	55 TR/VAL/TE	2Dp	U-Net++	42.85	15.07	0.87						
Zhou (162)	2022	Pelvis	13 TR, 2 VAL, 2 TE, 4-x CV	2Dp	U-Net			0.779 ± 0.069, 0.915 ± 0.055						Mean Surface Distance
Jiang (163)	2022	Body	50 tr/VAL, 11 TE	2Dp	CNN		35.31 ± 2.13	0.959 ± 0.012						RMSE
		Lung					35.59 ± 1.87	0.924 ± 0.020						
		Heart					37.89 ± 2.89	0.985 ± 0.005						
		Bone					33.07 ± 1.72	0.974 ± 0.004						
		Tumor					135.54 ± 1.57	0.980 ± 0.005						
Yoo (164)	2022	Brain	50 TR, 5 VAL, 10 TE	3Dp	CNN	5.79 ± 1.59	37.88 ± 2.15	0.98 ± 0.03		0.011 ± 0.003		96.2, 99.6		$\gamma_{1}$, $\gamma_{2}$
Deng (165)	2022	H&N	30 TR, 15 TE	2Du	GAN	140.7 ± 54.8	24.44 ± 3.7	0.964 ± 0.02						RMSE
		Pelvis	+ 14 TE			97.44 ± 16.6	27.86 ± 1.9	0.98 ± 0.01						
O (166)	2022	H&N	35 TR, 10 VAL, 15 TE	3D	GAN	79.4 ± 7.7				< 0.3 Gy	0.14 (0.34 to 0.06)	100.0 ± 0.1		PTV, $D_{95}$, $\gamma_{2}$
Xie (167)	2023	Breast	80 tr/VAL, 40 TE	2Du	GAN	50.34 ± 6.09	29.21 ± 1.60	93.72 ± 2.39				99.69 ± 0.22		$\gamma_{3}$
		Thorax				21.18 ± 3.76	30.02 ± 1.31	95.32 ± 1.67	f			98.98 ± 0.64		$\gamma_{2}$
Deng (168)	2023	H & N	44 tr/VAL, 15 TE	2Du	GAN	32.05 ± 9.23	27.80 ± 2.27	0.963 ± 0.018						RMSE, GMSD
		Pelvis				32.05 ± 9.23	27.80 ± 2.27	0.963 ± 0.018						
De (169)	2023	Pelvis	31 TR, 16 VAL, 9 TE	2Du	GAN	50 [19–30]	39.4 [39.0–40.8]	0.74 [0.68–0.79]						
Bladder					19 [13–29]	40 [39–45]								
Rectum					18 [12–23]	41 [37–45]								

CBCT scanners generate a cone-shaped x-ray beam that is incident on a flat panel detector, unlike CT scanners with fan-shaped x-ray beams and multi-slice detectors. The flat panel detector offers a wide coverage along the z-axis and high spatial resolution but also suffers from decreased signal due to scattered x-rays coming from the whole body. This results in significant quantitative CT errors as a result of severe streaking and cupping artifacts. When utilizing images for dose calculations, these errors introduce challenges in the calibration of Hounsfield Units (HU) to electron densities. HU represents the radiodensity of tissues in computed tomography (CT) scans.

CBCT can also suffer from degraded image contrast and bone suppression (170). As CBCT images are significantly degraded, they cannot be used for quantitative RT. CBCT Hounsfield Unit (HU) can be corrected and restored relative to CT using Deep learning (DL) based approaches, as shown in Table. The CBCT image is created through a combination of hundreds of projections in different directions. Before volume reconstruction, few studies applied neural networks to 2D projections, namely, the projection images. The CBCT volume was reconstructed from the improved quality projection images. Another approach relies on reconstructed CBCT images as inputs and produces sCT images with enhanced image quality. Utilizing projection domain methods for training with an extensive dataset of over 300 2D projection images offers the advantage of achieving a desired level of proficiency with a reduced number of training iterations compared to conventional image domain methods, which typically require approximately 100 iterations to achieve similar competence. CBCT images also suffer from artifacts such as cupping and streaking caused by scattering, whereas projection images are easier to learn for neural networks. Further, images have higher artifactual variation between patients, so much so that image domain methods rarely train models on non-anthropomorphic phantoms since the data collected is useless. However, in the projection domain, there is little variation in image features.

Therefore, Nomura et al. (171) showed that non-anthropomorphic phantom projections can also be used to learn to scatter distribution features that characterize anthropomorphic phantom projections. As a result, the neural network learned how to relate scatter distribution to objective thickness in the projection domain. Image scatter artifacts have a much more complicated relationship to objective appearance and cannot be easily learned. As the ground truth in the reviewed studies is often the corresponding CT images/projections from the same patient, CBCT images/projections are typically used while training. CT and CBCT are often out of geometric agreement, and registration reduces artifacts caused by the mismatch. As part of a pancreas study, Liu et al. (149) compared CBCT/CT training data rigidly and deformably registered. The researchers found that sCT created from rigidly registered training data produced lower noise and better organ boundaries compared with deformably registered CT (56.89 * 13.84 HU, P>0.05). As Kurz et al. (142) have demonstrated, generating sCT with satisfactory quality can be achieved without using pixel-wise loss functions in a cycle-GAN.

According to Hansen et al. (136) and Landry et al. (143), the registration step can be bypassed by correcting CBCTs first by conventional methods and then using the corrected CBCTs as ground truth. The corrected CBCTs do not require registration because the geometry of the corrected CBCTs remains the same as the original CBCT. However, CBCT generating methods in this setting limit the quality of sCT. Study findings suggest that DL-based methods have better image quality than conventional CBCT correction methods on the same datasets (96, 135, 137). They found that Adrian’s U-Net based method was more accurate and better suited to registering bone geometry than an image-based method or a deformable method. A comparison of Harms et al.’s (138) sCT to real CT study also demonstrated reduced noise and an improved subjective similarity. Corrective methods that are conventional are designed to improve only one specific aspect of image quality. DL-based methods, on the other hand, can modify every aspect of image quality to simulate CT, including noise level, which typically is not considered in conventional methods. Cycle-GAN outperformed both GAN and U-Net in several studies comparing the same patient datasets.

An analysis of 135 pelvic patients with 2.5D conditional GAN was conducted by Zhang et al. (148). Additional 15 pelvic and 10 H & N patients were analyzed afterwards. In both testing groups, the network predicted sCT at similar MAEs, showing that pre-trained models can be transferred to varying anatomical regions. In addition to different GAN architectures, the researchers compared U-Net configurations and found that it was statistically worse than any GAN configuration. The cycle-GAN has been tested with unpaired training in three works (141, 142, 144). A study performed the unsupervised training comparison of cycle-GAN, DCGAN (172), and PGGAN (173), where the first performed better in terms of image similarity and dose agreement.

The dosimetric accuracy of sCTs is significantly improved over that of original CBCTs, and an approach is used to calculate photon dose based on sCT. Select dose-volume histogram (DVH) metrics and dose or gamma differences have been investigated as a basis for evaluating sCT feasibility in VMAT planning at various body sites. According to Liu et al. (156) local dosimetric errors are large in areas with severe artifacts. These artifacts and dosimetric errors were successfully mitigated using sCT. With proton planning, it is more difficult to achieve acceptable dosimetric accuracy due to the range shift, which can be up to 5 mm (174).

### 3.3 PET attenuation correction

For PET Attenuation Correction (AC), the influence of synthetic Computed Tomography (sCT) error on PET quantification has been analyzed. It is extremely difficult to specify an error tolerance beyond which clinical decision making is affected; however, it has generally been accepted that quantitative errors of 10% or less rarely affect diagnostic imaging decisions (175). Most of the methods proposed in the studies met this criterion, based on their average relative biases. It should be noted however, that because of variations among study objects, there may be a bias exceeding 10% in some volumes of interest (VOI) for some patients (176, 177), suggesting that when interpreting results, it is important to take into account the standard deviation of the bias as well as the mean, since the proposed methods may not have good local performance for some patients. The results for this section is provided in Table 4. As an alternative to providing the mean and standard deviation in demonstrating the performance of the proposed methods, listing or plotting all the data points, or at least their range, would ultimately prove more useful (178). Being made up of high density and atomic number, bone has the most capacity for attenuation, and its accuracy on sCT has a huge impact on the final results of attenuation-corrected PET. It is more common for PET AC to evaluate the geometric accuracy of bone on sCT than radio therapy (RT). It has been shown that more accurate CT images generated by learning based methods result in more accurate PET AC (179–181).

**Table 4:** Overview of methods for PET Attenuation Correction.

Reference	Year	Tumor	Dataset	MRI	Deep Learning		Image Evaluation		PET-related		
					Config	Arc	MAE [HU]	DSC	tracer	PETerror [%]	Others
Liu (182)	2018	Body	100 TR, 28 TE	PET, no att corrected	2Dp	U-Net	111 ± 16	0.94 ± 0.01	${\;}_{18}^{\;}F-\mathrm{FDG}$	−0.6 ± 2.0	absolute error
Gong (180)	2018	H & N	32 TR, 8 TE, 5-x CV	3T Dixon	2Dp	U-Net	13.8 ± 1.4	076 ± 0.04	${\;}_{18}^{\;}F-\mathrm{FDG}$	< 3	
			12 TR, 2 TE, 7-x CV	± ZTE	2Dp	U-Net	12.6 ± 1.5	0.80 ± 0.04	${\;}_{18}^{\;}F-\mathrm{FDG}$	< 3	
Jang (183)	2018	Head	30p6 TR, 8 TE	1.5/3T UTE	2Dp	U-Net	0.76 ± 0.03		${\;}_{18}^{\;}F-\mathrm{FDG}$	< 1	
			6 TR, 8 TE				0.96 ± 0.01				
							0.88 ± 0.01				
Liu (184)	2018	Head	30 TR, 10, 5pet TE	1.5 T1 GRE Gd	2Dp	CNN		0.971 ± 0.005	n.a.		
					2Dp	CNN		0.936 ± 0.011			
					2Dp	CNN		0.803 ± 0.021			
Bradshaw (185)	2018	Pelvis	12 TR, 6 TE	3T T1 GRE T2 TSE	3Dp	CNN		0.99 ± 0.00	${\;}_{18}^{\;}F-\mathrm{FDG}$		RMSE
					3Dp	CNN		0.48 ± 0.21			
					3Dp	CNN		0.94 ± 0.01			
					3Dp	CNN		0.88 ± 0.03			
					3Dp	CNN		0.98 ± 0.01			
Dong (186)	2019	Body	80 TR, 39 TE	PET, no att corrected	3Dp	GAN	109 ± 19	0.87 ± 0.03	${\;}_{18}^{\;}F-\mathrm{FDG}$	< 1.0	NCC, PSNR, ME
Arabi (181)	2019	Head	40 TR, 2-x CV	3 T1 GRE	3Dp	GAN	101 ± 40	0.80 ± 0.07	${\;}_{18}^{\;}F-\mathrm{FDG}$	3.2 ± 3.4	rel vol dif, SUV
							302 ± 79			1.2 ± 13.8	surf dist ME,
							407 ± 228			3.2 ± 13.6	RMSE,
							10 ± 5			3.2 ± 13.6	PSNR, SSIM,
Blanc-Durand (177)	2019	Head	23 TR, 47 TE	3T ZTE	3Dp	U-Net		0.81 ± 0.03	${\;}_{18}^{\;}F-\mathrm{FDG}$	−0.2 ± 5.6	Jaccard index
Ladefoged (187)	2019	Head	60 TR, 19 TE, 4-x CV	3T m-Dixon UTE	3Dp	U-Net		0.90 ± 0.07	${\;}_{18}^{\;}F-\mathrm{FET}$		biol tumor, vol, SUV
Spuhler (188)	2019	Head	44 TR, 11 VAL, 11 TE	1.5 T1 GRE	2.5Dp	U-Net			${\;}_{11}^{\;}C-\mathrm{WAY}$	−0.49 ± 1.7	synt μ−map,
									${\;}_{11}^{\;}C-\mathrm{DASB}$	−1.52 ± 0.73	kin anal
Torrado (179)	2019	Pelvis	15 TR, 4 TE, 4-x CV	3T T1 GRE Dixon	2Dp	U-Net			${\;}_{18}^{\;}F-\mathrm{FDG}$	1.7 ± 2.0	μ−map
										1.8 ± 2.4	
										3.8 ± 3.9	
Armanious (189)	2020	Body	100 TR, 25 TE	PET, no att corrected	2.5Dp	GAN			${\;}_{18}^{\;}F-\mathrm{FDG}$	−0.8 ± 8.6	SUV ME
Gong (190)	2020	Head	32 TR, TE, 4-x CV	3T Dixonc	3Dp	GAN	15.8 ± 2.4%	0.74 ± 0.05b	${\;}_{18}^{\;}F-\mathrm{FDG}$	−1.0 ± 13	SUV
Baydoun (191)	2020	Thorax	14 TR, LOO	3 H Dixon	2Dp	GAN	67.45 ± 9.89		${\;}_{18}^{\;}F-\mathrm{NaF}$		PSNR, SSIM, RMSE
											
Chen (192)	2021	Brain	20 TR, 23 TE	3T $T_{1w}$	3Dp	CNN	62.07 ± 7.36	0.927 ± 0.015	${\;}_{18}^{\;}F-\mathrm{FDG}$	0.10 ± 0.56	SUV
Kläser (193)	2021	Head	20 TR, 23 TE	3T T1, T2	3Dp	CNN	110.98 ± 19.22		${\;}_{18}^{\;}F-\mathrm{FDG}$	4.74 ± 1.52	
Gong (194)	2021	Head	35 TR, 5-x CV	3 m-Dixon UTEc	2.5Dp	U-Net	10.94 ± 0.01%	0.87 ± 0.03b	${\;}_{11}^{\;}C-\mathrm{PiB}$, ${\;}_{18}^{\;}F-\mathrm{MK}-6240$	< 2	
Jiang (195)	2021	H & N	67 TR, 13 TE	3T $T_{1w}$	2Dp	GAN			${\;}_{18}^{\;}F-\mathrm{FDG}$		${43.64\pm 2.84}_{PSNR}$
											${0.981\pm 0.015}_{SSIM}$
Pozaruk (196)	2021	Prostate	18 TR, 10 TE	3T Dixon	2Dp	GAN			${\;}_{68}^{\;}\mathrm{GA}-\mathrm{PSMA}$	0.75 ± 0.64 max	SSIM,
										0.52 ± 0.62 mean	$\mu_{mapdiff}$
Ahangari (197)	2022	Body	15 TR, LOO	3T Dixon	3Dp	CNN	62		${\;}_{18}^{\;}F-\mathrm{FDG}$	−2.3	SUV
Li (198)	2022	Head	23 TR, 6 VAL, 5 TE		2Dp	CNN			${\;}_{18}^{\;}F-\mathrm{FDG}$	< 10	${25.63\pm 3.18}_{PSNR}$,${92.84\pm 2.69}_{SSIM}$
		Neck				CNN				average	${26.18\pm 3.39}_{PSNR}$,${89.83\pm 4.85}_{SSIM}$
		Chest				CNN					${25.81\pm 3.26}_{PSNR}$,${90.99\pm 4.85}_{SSIM}$
		Abdomen				CNN					${22.42\pm 3.27}_{PSNR}$,${90.37\pm 3.84}_{SSIM}$
		Pelvis				CNN					${30.31\pm 4.03}_{PSNR}$,${95.88\pm 1.90}_{SSIM}$
		Pelvis				CNN					RMSE, PCC, SUV
Olin (199)	2022	H&N	11 TR, LOO	3 T m-Dixon	2Dp	CNN			${\;}_{18}^{\;}F-\mathrm{FDG}$	0.6 ± 2.0	ME, RMSE
Shi (200)	2022	H&N	80 TR, 20 VAL, 85 TE		3Dp	U-Net	4.32 ± 0.74%		${\;}_{18}^{\;}F-\mathrm{FDG}$	< 1	RMSE, PSNR
Arabi (201)	2022	Torso	20 TR, 5 va/te, 5-x CV	3T m-Dixon	2Dp	CNN			${\;}_{18}^{\;}F-\mathrm{FDG}$	2.2 ± 1.6	SUV, p, DD, GPR
Ladefoged (202)	2023	Head	318 TR/VAL/TE	$T_{1w}$ m-Dixon	3Dp	U-Net	60 ± 35		${\;}_{18}^{\;}F-\mathrm{FET}$	< 1	SSIM, PSNR, BTV,
Rajagopal (203)	2023	Body	56 TR/VAL/TE	$T_{1w}$	3Dp	U-Net	0.066 ± 0.026		${\;}_{18}^{\;}F-\mathrm{FDG}$	< 7.6	SUV, RAE, SSIM

Deep learning (DL) based methods, designed to produce more precise sCT images, lead to enhanced accuracy in PET AC. Several studies have demonstrated the substantial improvements achieved by these methods. In contrast, PET AC using classical CT synthesis approaches exhibited an average bias of approximately 5% when compared to selected VOIs, while DL-based methods exhibited a reduced bias of around 2% in the same comparison (183, 184).

A 3D patch cycle-GAN was trained with unregistered MR/CT pairs, compared to atlas-based MRAC and CNNs with registered pairs by Gong et al. (190). A comparison of DL methods to atlas MRAC revealed that both performed better in DSC and MAE. CNN and cycle-GAN did not differ significantly in their performance in DSC and MAE. According to their research, cycle-GAN is able to avoid the challenge of training on perfectly aligned datasets, but more data is needed to improve its performance.

It was examined whether different network configurations (VGG-16 (48), VGG-19 and ResNet (44)) can be used as a benchmark with a 2D conditional GAN that receives either two Dixon inputs (water and fat) or four Dixon inputs (water, fat, in-phase, and opposed). When four inputs are used in the GAN, results are more accurate than the VGG-19 and the ResNet.

Several authors have proposed that the sCT could be obtained directly from diagnostic imaging, $T_{1}$- or $T_{2}$-weighted, by using standalone MRI scanners(32, 184) or hybrid machines (185).

Bradshaw et al. (185) a three CNN trained on Gradient Echo (GRE) and Turbo Spin Echo (TSE) MRI sequences, specifically, the $T_{1}$ and $T_{2}$ sequences. The CNN was trained to predict tissue segmentation across distinct classes, including air, water, fat, and bone. Subsequently, the model’s performance was compared with the default Magnetic Resonance Attenuation Correction (MRAC) method commonly employed by scanners. PET reconstruction had substantially lower RMSE when calculated with DL method and $T_{1}$/$T_{2}$ input. Recent studies have investigated a CNN with input either $T_{1}$ or Dixon and multiple echo UTE (mUTE) on a brain patient cohort, and found that it outperformed the others. A CNN was trained on 1.5 T $T_{1}$ diagnostic GRE data of 30 patients in Liu et al. (184). A total of ten patients from the same cohort were used and their results are reported in the following table. Using a 3 T MRI/PET scanner, they then predicted the pathology for five patients ( $T_{1}$ GRE), and calculated the error ($PET_{[}error]$), resulting in a 1% error rate. The authors concluded that DL-based approaches are flexible and suitable for handling heterogeneous datasets acquired using many scanner types.

### 3.4 Low dose CT to full dose synthetic CT (sCT)

The data-driven approach to automatically learning image features and model parameters makes deep learning (DL) an attractive option for low-dose Computed Tomography (LDCT) restoration. The existing literature primarily discusses two approaches for LDCT image enhancement. The results for this section is provided in Table 5. Some methods focus on direct image translation from LDCT to full-dose CT (FDCT), while others involve a two-step process. In the latter approach, DL is utilized to restore the sinogram, followed by image reconstruction using Filtered Back Projection (FBP). The proposed method by Dong et al. (204) reduces lower-resolution edges of objects with better down-sampling artifacts than an image-based one.

**Table 5:** Overview of methods for low-dose CT to high-dose CT generation.

Author	Year	Tumor	Dataset	Network	Low-dose scheme and fraction of full dose CT	Findings	Mode
Kang (205)	2018	Abdomen	8 TR, 1 TE	AE (ResNet)	Low mAs: 1/4 of full dose	PSNR (dB): 38.70	Image
Yi (206)	2018	Abdomen	708 s TR, 142 s TE	GAN	Low mAs: 5% of full mAs	PSNR (dB): about 34	Image
Shan (207)	2018	Abdomen	5 TR, 5 TE	GAN	Low mAs: 1/4 of full dose	PSNR(dB): 30.137 ± 1.938	Image
You (208)	2018	Abdomen	10, LOO	GAN	Low mAs: 1/4 of full dose	PSNR (dB): 25.372	Image
Han (209)	2018	Abdomen	8 TR, 1 VAL, 1 TE	U-net	Sparse view: 1/12 of full views	PSNR (dB): 40.4856	Image
Yang (210)	2018	Abdomen	4000 s TR, 2000 TE	GAN	Low mAs: 1/4 of full dose	Validated in double-blinded reader study	Image
Liu (211)	2018	Whole body	300 s TR, 50 s TE	U-net (Encoderdecoder)	Low mAs: fraction not specified	PSNR (dB): 42.3257	Image
Shan (173)	2019	Abdomen & chest	10 TR, 60 TE	U-net	Low mAs: about 1/3 to 1/8 of full dose	Validated in double-blinded reader study	Image
Zhao (212)	2019	Chest	3 TR, 3 TE	AE	Low mAs: 3% of full mAs	PSNR (dB): about 22	Image
Lee (213)	2019	Chest	7 TR, 8 TE	U-net	Sparse view: 1/4 of full views	PSNR (dB): (42.73, 52.14)	Projection
Dong (204)	2019	Head	200 s TR, 100 s TE	U-net	Sparse view: 1/12 of full views	PSNR (dB): 37.21 for sparse view	Projection
Wang (214)	2019	Head	30, 5x CV	CycleGAN	Low mAs: 0.5% of full mAs	NMSE (%): 1.63 ± 0.62	Image
Li (215)	2020	Abdomen	1382 TR, 1345 va/te	WGAN	Low mAs: 1/4 of full dose	PSNR: 22.27, SSIM: 0.78	Image
Chi (216)	2020	Abdomen	4036 TR, 296 TE	LSGAN	Low mAs: 1/4 of full dose	PSNR: 44.40, SSIM: 0.98	Image
Ma (217)	2020	Abdomen	2378 TR, 211 TE	LSGAN	Low mAs: 1/4 of full dose	PSNR: 32.70, SSIM: 0.91	Image
Yin (218)	2021	Lung	2400 s TR, 100 s TE	GAN	noise surpressed maintaining the structure and edge details	PSNR: 29.6957, SSIM: 0.6916	Image
Gu (219)	2021	Chest	36535 s TR, 13530 TE	CycleGAN	Low mAs: 1/4 of full dose	PSNR: 30.87, SSIM: 0.66	Image
Jiang (220)	2022	Lung	203 TR/VAL/TE	CNN	Low mAs: 4.6% & 9.2% of full dose	MAE: 51 HU ± 4	Image
Zhu (221)	2022	Abdomen	1024 s TR, 256 s TE	CNN (SMU-Net)	Low mAs: 1/4 of full dose	PSNR: 50.47 ± 6.78, SSIM: 0.99 ± 0.0067	Image
Zhou (222)	2022	Abdomen	10 TR, 7 TE	GAN(TTSR)	Low mAs: 1/4 of full dose	PSNR: 31.16 ± 1.38, SSIM: 0.73 ± 0.06	Image
Yang (223)	2023	Abdomen	2378 s from 10	CNN(MDAM)	Low mAs: 1/4 of full dose	PSNR: 29.26 ± 1.60, SSIM: 0.87 ± 0.055	Image
Yang (223)	2023	Abdomen	104	CNN(MDAM)	Low mAs: 10% of full dose	PSNR: 36.06 ± 1.14, SSIM: 0.95 ± 0.008	Image
Gao (224)	2023	Abdomen	5410 TR, 526 TE	CNN(ADBNET)	Sparse view: 1/4 of full dose	PSNR: 38.82 ± 0.19, SSIM: 0.92 ± 0.002	Projection
Li (225)	2023	Abdomen	8 TR, 2 TE	CNN(PCCNN)	Low mAs: 1/4 of full dose	PSNR: 30.67 ± 0.12, SSIM: 0.91 ± 0.001	Image
			+ 10 TR		Low mAs: 1/4 of full dose	PSNR: 32.05 ± 0.10, SSIM: 0.93 ± 0.001	Projection

There is a possibility that projection-based methods do not directly detect prediction errors, while image-based methods do. During the reconstruction process, the predicted error on the sinogram will be compensated for, and the outcome will be the average of all the sinograms. These models are more error-resistant because of their projection-based nature. The network may be encoded with a mapping from polar to Cartesian coordinates for direct mapping from the projection domain to the image domain.

In their progressive method, Shan et al. (173) generated a sequence of denoised images at different levels of noise by iteratively denoising the input LDCT. Rather than directly mapping LDCT or FDCT images, Kang et al. (226) mapped their wavelet coefficients. Better recovery of structures was achieved with wavelet transformations compared to direct mapping. DL-based methods are less time consuming than iterative reconstruction methods and do not require prior knowledge of energy spectrum. The LDCT model reported by Wang et al. (214) was trained on an average personal computer in 1 minute and generated an entire 3D volume from denoised images. Due to the resource-intensive nature of traditional iterative reconstruction methods, their implementation is limited on personal computers, especially when slice thickness and field of view (FOV) are small.

Numerous studies have conducted comparisons between traditional iterative methods and state-of-the-art DL-based techniques. Among these advanced methods, Total Variation (TV) regularization has received attention. TV-based techniques, while known to sometimes over smooth images and create uneven textures, excel in preserving fine structures and maintaining image texture similarity to FDCT scans. Utilizing analytical optimization objectives in deep learning enhances image quality while preserving texture, resulting in predictions that closely align with the ground truth, as represented by FDCT images. This improvement is quantitatively measured through metrics such as peak signal-to-noise ratio (PSNR), structural similarity (SSIM), and mean absolute error (MAE). A double-blinded reader study conducted by Shan et al. (173) proved their DL-based method performed similarly to three commercially available iterative algorithms for noise suppression and structural fidelity. Almost all the studies reviewed used their restored FDCT images for diagnostic purposes. This method is particularly suitable for adaptive RT where re-scanning and planning throughout a treatment course is common, as Wang et al. (214) evaluated it in the context of RT treatment planning. Planning CT requires accurate Hounsfield Unit (HU) and dose calculation accuracy vs diagnostic CT, which emphasizes high resolution and low contrast. When a dose of 21 Gy is prescribed, the average difference in dose volume histogram (DVH) metrics between original FDCT and synthetic FDCT is less than 0.1 Gy (P>0.05). Although the training and testing strategies may differ among these studies, the results are similar. Most of the reviewed studies used the dataset from the AAPM 2016 LDCT Grand Challenge (227). Because LDCT does not contain any clinical data, it is also used as an example of Poisson noise or a downsampled sinogram in many other studies. There are a few exceptions, such as Yi et al. (206) who used piglets, and Shan et al. (173) who used LDCTs from real patients. It is therefore important to evaluate these methods against actual LDCT datasets since simulated noise may not accurately reflect the properties of true noise and potential artifacts.

## 4 Discussion

In many domains of biomedical research and clinical treatment, imaging has become a necessary component. Radiologists identify and quantify tumors from Magnetic Resonance Image (MRI) and Computed Tomography (CT) scans, and neuro-scientists detect regional metabolic brain activity from Positron Emission Tomography (PET) and functional MRI scans. Biologists study cells and generate 3D confocal microscopy data sets, virologists generate 3D reconstructions of viruses from micrographs, radiologists identify and quantify tumors from MRI and CT scans, and neuro-scientists detect regional metabolic brain activity from PET and functional MRI scans. In contrast to traditional digital image processing and computer vision approaches that need many MRI modalities to properly show all areas. There are few novel Deep Learning (DL) approaches available (discussed in literature) for generating brain sCT images that only requires one MRI pulse sequence to accurately display all regions (43, 228, 229).

DL-based image synthesis is a young and rapidly developing field, with all of the studies evaluated published within the past five years. There is much literature on DL-based image synthesis. Future studies need to address specific unanswered questions. Since GPU memory constraints prevented training on three-dimensional (3D) slices, some DL algorithms were trained on two-dimensional (2D) slices. Unlike 3D loss functions, 2D loss functions do not consider continuity in the third dimension, thus making slices appear discontinuous. In addition to using 3D patches to train models that exploit 3D spatial information more effectively, they can also extract features from larger-scale images (34, 81). A 2D and 3D model was examined using the exact U-Net implementation by Fu et al. (73). The study’s findings suggest that a 3D sCT offered more accurate results with smaller MAE. In the absence of additional data, the model might use many adjacent slices to gather additional 3D context or generate independent networks for each of the three orthogonal 2D planes (230).

A DL-based approach can produce images that are more realistic improve quantitative metrics. Depending on the technology, it can take from an hour to days to train a model using DL-based approaches. A synthetic image for a new patient can be generated within seconds or minutes after training a model. Our study reviews the feasibility of using various imaging methods to build CTs using DL-based methods. It has become possible to train large datasets and translate images in seconds due to higher computing capabilities. DL’s clinical applications are made simpler by fast image-to-image translation, proving the method’s usefulness.

1. MR based RT: There are many types of sCT generation approaches, but MR only RT with DL is the most prevalent. The eighty two studies in this review demonstrate that DL algorithms effectively produce sCT from MRI data. Many methods of training and combinations have been proposed. The pelvis and the head and neck can be treated using photon radiotherapy (RT) and proton therapy, which achieve high image similarity and dosimetry accuracy. As part of the feasibility phase of testing, application of DL algorithms to abdominal and thoracic positions with significant motion are showing promise (37, 41, 59, 60, 86, 89, 104, 116–118, 185, 231). The MR-only simulation of pediatric patients could be extremely beneficial when their simulations are repeated since they are more radiation-sensitive than adults.
  It is necessary to confirm the geometrical accuracy of sCT before it can be used for clinical planning, mainly if MRI or sCT is used to replace CT for position verification. So far, research on DL-based sCT has been limited to a few studies. There have only been two studies that used CBCT and digitally reconstructed radiography to assess their alignments: Gupta et al. (33) for brain cancer and Olberg et al. (41) for breast cancer. The accuracy of sCT produced with standard 3T techniques has been extensively investigated, notably for geometric accuracy. Research is critical to enhancing the clinical application of sCT (232–234).
  DL-based sCT generation may reduce the duration of treatment in MR-guided RT, (235–239) because solitary MRI allows daily image guidance and plan modification. It is essential to assess the accuracy of dose calculation in a magnetic field before using it clinically. The current state of research on this topic is limited to studies on abdominal and pelvic tumors (59) and they have only considered low-strength magnetic fields. Recently, Groot Koerkamp et al. (240) reported the first dosimetric study demonstrating DDs for breast cancer patients treated with DL-based sCT. It is encouraging that the results were positive, but we recommend further study of other anatomical sites and magnetic field strengths.
2. CBCT to CT: CBCT imaging is an integral part of the daily patient setup for photon and proton RT. Due to scattering and reconstruction abnormalities, it is not routinely used to adjust daily plans and recalculate doses. This problem can be addressed in several ways (241), including image registration (242), scatter correction (243), a look-up table to rescale HU intensities (244), and histogram matching. In contrast to image registration and analytical adjustments, converting CBCT to sCT enhanced image quality. CBCT-to-CT conversion presents a challenge for clinical use because of the two imaging technologies’ different fields of view (FOV). This is usually overcome by cropping, registering, and resampling the volume to a smaller CBCT size than planned.
  However, the small field of view presents some challenges. For missing information (145), some have suggested assigning water equivalent density to the CT body contours. The sCT patch can also be sewed directly to the intended CT, guaranteeing that the whole dose volume will be covered. This stage is essential for online adaptive RT, especially in areas with a high degree of motion, as Liu et al. hypothesized in their work on pancreatic cancer (149). There is currently no consensus on whether improving CBCT quality with synthesis and reconstruction is the optimal approach. In preliminary experiments, training convolutional networks for reconstruction resulted in greater generalizability to diverse anatomy.
3. PET attenuation Correction (AC): sCTs generated in this category are derived either from MRIs or PETs that have not been corrected. Attenuation maps in MRI/PET hybrid acquisitions are currently inaccurate due to limitations in attenuation map construction. DL-based sCT has always been more consistent than commercially available MRAC. This review suggests that using deep learning for synthetic CT (sCT) can overcome most of the challenges associated with current AC methods. Although there has been a consistent number of studies in this field over the past few years, the specific factors and trends in these studies vary. These studies focus primarily on translating images into CT. Alternatively, Shiri et al. (245) studied the most significant number of patients to date (1150 patients split into 900 pieces of training, 100 validations, and 150 testings). This field could benefit from DL’s direct-map prediction capabilities in the future.

## 5 Trends in deep learning

### 5.1 Application

Deep Learning (DL) approaches, including supervised, semi-supervised, unsupervised, and reinforcement learning may tackle a wide range of issues. Computer vision and digital image processing applications have been divided into three groups by some researchers: structural scenarios, non-structural scenarios, and miscellaneous application situations. The term “structural scenario” refers to a circumstance in which data is processed in relational structures that are clear, such as physical systems and chemical structures. The term “non-structural scenario” refers to a circumstance in which data is not structured, e.g., images and texts with ambiguous patterns.

For clinicians who manage the search for representative images, it does not matter that how many times the data is reproduced. CT scanning, ultrasound, and MRI are all used in x-ray imaging. Physicians may examine the body’s obscure or concealed third dimension in this manner.

#### 5.1.1 Image registration

Synthetic images can be used for diverse tasks downstream, revealing many possibilities. Intricate processes like image registration can be simplified with synthetic images generated using cutting-edge techniques. Chen et al. (192) have demonstrated in their work that synthetic images can facilitate streamlined workflows when it comes to registration by acting like reliable substitutes for real-world images in streamlined workflows.

Image alignment is essential for cross-domain image registration, where synthetic image generation is used to create images. In feature-based supervised registration of 3D multimodal images, deep learning has been used in several ways. To predict registration parameters, researchers have primarily used deep regression models (246–248). As well as being used in pre-processing, deep learning has also been used in the process of determining control points, which are then used to determine the registration parameters based on the information that is acquired from the deep learning technique.

An AIRNet (affine image registration network) model was developed by Chee and Wu (249) to predict the parameters of affine transformations between 2D and 3D images. An intra-patient T1 and T2 MRI image of the head was transformed using a deep learning regression model by Sloan et al. (250). According to Liu et al. (251), multi-modal medical image registration can be performed using synthetic image generation and deep learning. For rigid-body medical image registration, Zou et al. (252) implemented feature extraction and interest/control points-based deep learning models.

CT synthesis using MR-based technology also proves to be promising for radiation treatment planning and PET attenuation correction. In deformable registration when significant geometric distortion is allowed, direct registration between CT and MR images is even less reliable because of disparate image contrast. By replacing MRI with synthetic CT images, McKenzie et al. (253) reduced an inter-modality registration problem to intra-modality registration in the head and neck by using a CycleGAN-based method. CBCT technology is being increasingly adopted to improve the quality of radiation therapy, including higher diagnostic accuracy and better auto-contouring based on improvements in image registration, deformable image registration (DIR) and simulator analysis of CT images (133, 134). These capabilities are being offered in an increasing number of applications.

#### 5.1.2 Image augmentation

A synthetic image can also enhance training sets in supervised learning applications. As Frid et al. (254) demonstrated, synthesized data augmentation can be a productive tool for improving model performance and robustness, which is one of the critical challenges of training deep learning models on limited datasets. In addition to extending synthetic images in downstream tasks across a broad range of domains, these investigations also shed light on the transformative role that synthetic images can play in optimizing complex processes in diverse domains.

A method for increasing the size of existing databases is known as data augmentation. A synthetic set of data is typically generated from the original database data. A synthetic image is created from the original dataset by using a particular method and generating a certain number of synthetic images from it. The former question has given rise to numerous methods, many of which are aimed at addressing it, including generative adversarial networks (255), random cropping (256), geometric transformations (257, 258), mixing images (259), and neural style transfers (260).

To improve the network’s generalizability and reduce overfitting, data augmentation is heavily used in deep neural network training nowadays. There are currently no data augmentation operations that can cover all variations of the data, as they are all manually designed operations, such as rotation and color jittering. The search space of Cubuk et al. (261) was still restricted to basic handcrafted image processing operations when they proposed to learn an augmentation policy with reinforcement learning. As a result, GANs are much more flexible for augmenting the training data, as they can sample the whole distribution of data (262). In styleGAN, realistic face images can be generated with unprecedented detail. Using this technique, images of pathology classes with sufficient numbers of cases could be generated from chest x-ray datasets. Medical data distribution is well known to be highly skewed with common diseases accounting for the majority of data. Rheumatoid arthritis, sickle cell disease, and other rare diseases cannot be adequately trained. The long tail of these diseases can be detected by radiologists. It is also anticipated that GANs will be used for the purpose of synthesizing cases and circumstances with uncommon pathologies. This will be done by conditionally generating information with medical experts supplying the conditioned information either on the basis of text descriptions or drawings.

#### 5.1.3 Datasets, open-source libraries and tools

Computer vision techniques are evaluated using a variety of datasets and standards in different branches, including medical imaging (healthcare), agriculture, surveillance, sports and automotive etc. The implementation of DL in computer vision (medical imaging) is limited by a relatively small training dataset and a huge imaging volume. Example datasets include CT medical images (CT images from cancer imaging archive with contrast and patient age), Deep Lesion (contains 32,120 axial CT slices from 10,594 CT scans of 4,427 unique patients), OASIS Brain (Open Access Series of Imaging Studies dataset for normal aging and Alzheimer’s Disease), MRNet (dataset consists of 1,370 knee MRI) and IVDM3Seg (3D multi-modal MRI datasets of in-vitro diagnostics of the lower spine). Some open-source libraries have been established by certain research organizations and researchers, which comprise both common and classic computer vision techniques e.g., OpenCV, SimpleCV and TensorFlow etc (176, 263, 264).

MIPAV (Medical Image Processing, Analysis, and Visualization) is a java-based tool that allows for quantitative analysis and visualization of medical images from a variety of modalities, including PET, MRI, and CT. FSL (FMRIB Software Library) encompasses an extensive array of analysis tools designed for processing FMRI, MRI, and DTI brain imaging data (265). AFNI (Analysis of Functional Neuro Images) is a Python-based application that analyzes and displays data from different MRI modalities, including anatomical, functional MRI (FMRI), and diffusion weighted (DW) data (176, 263, 264).

#### 5.1.4 Predictive analytics and therapy using computer vision

The use of computer vision in surgery and the treatment of certain illnesses has demonstrated to be quite beneficial especially in the field of surgery. Three-dimensional (3D) modeling and rapid prototyping technologies have lately helped medical imaging modalities such as CT and MRI. Human activity recognition (HAR) is also one of the most well-studied computer vision challenges. S. Zhang et al. (266) provide an overview of several HAR techniques as well as their evolution with traditional Chinese literature.

In vision-based activity recognition, the authors emphasize developments in image representation methodologies and classification algorithms. Common representation approaches include global representations, local representations, and depth-based representations. They divide and describe human activities into three levels, in that order: action primitives, actions/activities, and interactions. They also offer a description of the HAR application’s classification techniques (266, 267).

### 5.2 Diffusion models

Developing realistic and high-fidelity images is a challenge that has seen a paradigm shift with the emergence of diffusion models. Intuitive patterns and dependencies within image data can be captured using these probability distribution models based on probability distributions. According to recent studies, diffusion models can produce diverse and realistic samples more effectively than traditional generative models, as demonstrated in work by Dhariwal et al. (268). Diffusion models are robust and versatile tools for image synthesis since they can consider the underlying uncertainty in pixel values. This article intends to shed light on the potential of diffusion models to redefine the landscape of image synthesis in various domains, drawing inspiration from recent developments and applications in multiple fields.

Integrating diffusion models can profoundly advance diagnostic and therapeutic applications of diffusion models in medical imaging. According to Hung et al. (269), diffusion models can capture nuanced variations in medical images, enhancing the realism of synthesized medical data. This article aims to demonstrate how diffusion models can be used to address challenges like data scarcity and to create realistic synthetic datasets based on image synthesis. Utilizing diffusion models is a critical trend in medical imaging as synthesis data is increasingly used for training machine learning models, resulting in improved diagnostic accuracy and treatment planning. This article examines diffusion models in the context of current advancements and future possibilities in medical imaging.

### 5.3 Open issues

There are studies in medical imaging research that demonstrate accuracy of above 95%. Though, we are concerned with more than simply the accuracy of a classifier. Because false negatives and false positives in medical imaging may have catastrophic effects. This is one of the reasons why, despite their high performance, stand-alone decision systems are not widely used. In this section, we will describe many potential research topics and open concerns for computer vision in medical imaging.

Imaging Modality: Medical imaging modalities are classified according to how images are generated. In radiology, a modality is a phrase that refers to a certain kind of imaging, such as CT scanning, ultrasound, radiation (x-rays), and MRI. X-ray machines, which are made up of a single x-ray source and produce two-dimensional images, are examples of radiation-generated images. In literature, medical imaging modalities algorithms have received a great attention, but it is critical that the medical imaging modalities algorithms, be designed to retain high performance.

Generative Medical Image Synthesis: Inspired by the GAN, because of its capacity to generate data without explicitly modeling the probability density function, GANs have gotten a lot of interest in the computer vision field. If diagnostic images are to be utilized in a publication or put into the public domain, patient’s agreement could be necessary, depending on institutional rules. GANs are commonly used in the medical imaging for image synthesis. This helps to address the privacy concerns around diagnostic medical images as well as the lack of positive instances for each disease. Another barrier to the implementation of supervised training techniques is the lack of professionals who can annotate medical images (52).

Interpretability/Explainability in Medical Image Analysis: An explanation of the machine learning (ML) algorithm can be described as interpretability. Various computer vision algorithms achieve outstanding results at the cost of greater complexity. As a result, they become less interpretable, perhaps leading to distrust. DL-based approaches, have shown to be quite successful for several medical diagnostic tasks, outperforming human specialists in certain cases. However, the algorithms’ black-box nature has limited their clinical use. Recent explainability studies have attempted to demonstrate the characteristics that have the most impact on a model’s choice. Furthermore, interpretability findings are often based on a comparison of explanations with domain knowledge. As a result, objective, quantitative, and systematic assessment procedures are required (270). Finally, AI safety in healthcare is intimately linked to interpretability and explainability.

## 6 Conclusion

This study includes a broad overview of computer vision techniques as well as a complete assessment of medical imaging with respect to CT, CBCT, PET and MRI techniques. We looked at current digital image processing techniques with respect to medical imaging. We did our best to emphasize both the potential and the obstacles that this medical imaging application industry faces in the healthcare field. Our goal is to uncover the important need for computer vision algorithms in the clinical and theoretical context of medical imaging. This special research article discusses a few recent advancements in computer vision related to medical images and clinical applications.

In conclusion, this study presents a glimpse of computer vision in healthcare applications using medical images. Hopefully, future computer vision, analysis techniques, and ML of medical images will benefit from this paper. However, even though these works outperform conventional and state-of-the-art approaches, there are still limitations and challenges for computer vision and different algorithms and processing techniques of medical images. In addition, we discuss some potential future research areas in the sCT generation. We really hope that this survey proves to be useful. We believe that this survey will aid scholars and practitioners in their computer vision, medical imaging and related research and development.

## Data Availability

The original contributions presented in the study are included in the article/supplementary material, further inquiries can be directed to the corresponding author/s.
